# A comprehensive perspective of traditional Arabic or Islamic medicinal plants as an adjuvant therapy against COVID-19

**DOI:** 10.1016/j.sjbs.2023.103561

**Published:** 2023-01-13

**Authors:** Shabina Ishtiaq Ahmed, Sehrish Jamil, Humaira Ismatullah, Rashid Hussain, Shabana Bibi, Mayeen Uddin Khandaker, Aisha Naveed, Abubakr M. Idris, Talha Bin Emran

**Affiliations:** a Department of Plant Biotechnology, Atta-Ur-Rahman School of Applied Biosciences (ASAB), National University of Sciences and Technology (NUST), 44000, Islamabad, Pakistan; b The Standard College for Girls, 3/530 Paris Road, Sialkot Pakistan; c School of Interdisciplinary Engineering & Sciences (SINES), National University of Sciences and Technology (NUST), 44000 Islamabad, Pakistan; d Department of Biosciences, Shifa Tameer-e-Millat University, Islamabad, Pakistan; e Yunnan Herbal Laboratory, College of Ecology and Environmental Sciences, Yunnan University, Kunming 650091, China; f Center for Applied Physics and Radiation Technologies, School of Engineering and Technology, Sunway University, Bandar Sunway 47500, Selangor, Malaysia; g Caribbean Medical University, Willemastad, Curacao-Caribbean Island, Curaçao; h Department of Chemistry, College of Science, King Khalid University, Abha 62529, Saudi Arabia; i Research Center for Advanced Materials Science (RCAMS), King Khalid University, Abha 62529, Saudi Arabia; j Department of Pharmacy, BGC Trust University Bangladesh, Chittagong 4381, Bangladesh; k Department of Pharmacy, Faculty of Allied Health Sciences, Daffodil International University, Dhaka 1207, Bangladesh

**Keywords:** Traditional Arabic or Islamic medicinal plants, COVID-19, Phytochemicals, ACE2, Antiviral, Adjuvant therapy, SARS-CoV-2, Aleo vera

## Abstract

COVID-19 is a pulmonary disease caused by SARS-CoV-2. More than 200 million individuals are infected by this globally. Pyrexia, coughing, shortness of breath, headaches, diarrhoea, sore throats, and body aches are among the typical symptoms of COVID-19. The virus enters into the host body by interacting with the ACE2 receptor. Despite many SARS-CoV-2 vaccines manufactured by distinct strategies but any evidence-based particular medication to combat COVID-19 is not available yet. However, further research is required to determine the safety and effectiveness profile of the present therapeutic approaches. In this study, we provide a summary of Traditional Arabic or Islamic medicinal (TAIM) plants' historical use and their present role as adjuvant therapy for COVID-19. Herein, six medicinal plants *Aloe barbadensis Miller, Olea europaea, Trigonella foenum-graecum, Nigella sativa, Cassia angustifolia,* and *Ficus carica* have been studied based upon their pharmacological activities against viral infections. These plants include phytochemicals that have antiviral, immunomodulatory, antiasthmatic, antipyretic, and antitussive properties. These bioactive substances could be employed to control symptoms and enhance the development of a possible COVID-19 medicinal synthesis. To determine whether or if these TAIMs may be used as adjuvant therapy and are appropriate, a detailed evaluation is advised.

## Graphical abstract

**Figure:**
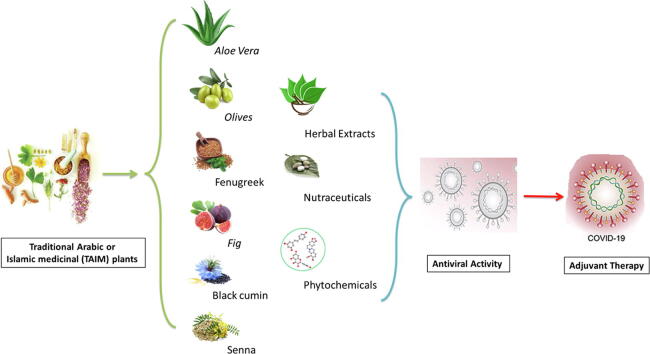

## 1 Introduction

In human, corona virus was isolated from a patient's respiratory tract suffering from common cold and named as B-814. Morphologically, the name corona describes the crown-like look of the surface. Coronaviruses are categorized into Alpha, Beta, Gamma and delta CoV (Zhu et al., 2020). Coronaviruses are RNA enveloped viruses and are member of family coronaviridae, of order Nidovirales. Coronaviridae is main cause of many diseases in mammals and birds such as common cold in fowl and fatal human pulmonary illness including bronchitics, pneumonia. Over the last 20 years, Coronavirus has led two major pandemics: SARs (severe acute respiratory syndrome) in 2002 caused 774 death from 8096 cases, and MERs (Middle East Respiratory Syndrome) in 2012 in Saudi Arabia (De Wit et al., 2016). MERs infected people with high mortality rate and caused 590 deaths from 1651 laboratory confirmed cases (Omrani et al., 2015). Chinese horseshoe bats and civets were considered the natural reservoir for SARs-CoV (Cui et al., 2019).

In December 2019, first victim of coronavirus was notified in China. After careful examination of respiratory samples, the virus strain was declared novel and initially named as 2019n-CoV (Zhu et al., 2020). Similarly, the Pneumonia epidemic occurred in 2020, resulted by a newly discovered corona virus. The virus was termed SAR-CoV-2 by the International Committee on Taxonomy of viruses (Zu et al., 2020). Both SAR-CoV-2 and 2019n-CoV belongs to beta genus (Li, 2016). 2019n-CoV and SARS-CoV-2 have some resemblance in genomic organization. More than 7736 confirmed cases were reported in China and the infection was detected in 18 countries. WHO declared a Public Health Emergency of International concern (PHEIC) for 2019n-CoV outbreak (World Health Organization (WHO), 2005). Whereas, in March 2020, the WHO database confirmed 574444 corona cases from 201 countries worldwide. Italy was one of the most affected countries, with more than 9000 deaths, then United state with 1243 deaths and then China with 3300 death (Chen et al., 2020, Dennison Himmelfarb and Baptiste, 2020).

SARS-CoV-2 encodes multiple non-structural and structural proteins. Structural proteins play important in the viral pathogenesis of COVID-19. Structural proteins of SARS-CoV-2 includes spike protein, membrane protein, envelope protein and nucleocapsid protein (Ahmed et al., 2020). These proteins are involved in the entry, attachment, fusion and assembly of viral particle which in turn responsible for the replication of virus. Therefore, these structural proteins may serve as potential molecular targets to develop vaccines and drugs against the SARS-CoV-2.

SARS-CoV-2 entry into the host cell is mediated through a 150 KDa transmembrane spike protein of the virus (Guo et al., 2020). It comprises two functional subunits, which mediates the binding to the host cell receptor angiotensin-converting enzyme 2 (ACE2) and fusion of viral membrane with the host cell. It has been reported that SARS-CoV-2 spike protein shared approximately 72 % of sequence identity with the SARS-CoV (Walls et al., 2020). The research also revealed that diversity in the sequence is responsible for the presence of unique fusion like cleavage site in SARS-CoV-2 which was absent in SARS-CoV (Coutard et al. 2020). Structural and biophysical analysis showed that, as compared to SARS-CoV, SARS-CoV-2 binds with the host cell receptor ACE2 receptor with more than 10-fold higher affinity (Wrapp et al., 2020). This binding results in the faster transmission and progression of SARS-CoV-2 in humans than SARS-COV (Wang et al., 2020a, Wang et al., 2020b).

Common symtoms of the virus includes high body temperature, cough, breathless, headache, diarrhoea, scratchiness in throat, and body soreness in COVID-19 patients (Huang et al., 2020, Wang et al., 2020a, Wang et al., 2020b). Usually, the virus spreads to human by human transmission (Phan et al., 2020). Various methods were used to manufacture SARS-CoV-2 vaccine including synthetic and physical procedures like methanal, ultraviolet radiation, beta-propiolactone. Similarly, virus with reduced pathogenicity, such as anti-inflammatory cytokine levels, and attenuated-viral vaccines can be created. This may lower the neutrophil influx and cause less lungs injury (Shang et al., 2020).

Furthermore, vaccine can be designed using S1-receptor binding domain (RBD), full-length spike protein, or expression in Virus Like Particles (VLP), DNA, or viral vectors. Spike protein-based vaccinations are speculated to create antibodies within the body and prevent viral genome uncoating and receptor binding. BNT162b1 and BNT162b2 are mRNA vaccines with nucleotide modifications. BNT162a1 is a urideine mRNA-based vaccination, while BNT162c2 is a vaccine based on self-amplifying mRNA33. SARS-CoV-2 RNA vaccines are being developed by BioNTech/Pfizer and Moderna. BNT162b1, BNT162b2, BNT162a1, and BNT162c2 are the four candidates for BioNTech/mRNA Pfizer's vaccine. The BNT162b1 codes the S protein's trimerized RBD. BNT162b2 encodes the full-length spike protein. In order to stabilise the spike protein in its pre-fusion state, proline was substituted for amino acids 986 and 987, resulting in the prefusion spike trimer encoded by Moderna's mRNA-1273 vaccine. Upon entering in the cell, the nucleotides of virus mRNA changed not only to increase half-life and translation, but also to prevent interferon-associated genes from being triggered (Jackson et al., 2020). Moreover, Aivita Biomedical developed AV-COVID-19, an analogous of nerve cell vaccine filled with SARS-CoV-2 antigens (De Wit et al., 2016). BacTRL-Spike, a Bifidobacterium vaccine tailored to carry artificial plasmid DNA encoding SARS-CoV-2 spike protein, was created by Symvivo Corporation (Zhou et al., 2020a).

COVID-19′s rapid transmission and asymptomatic spread demonstrates the need for an effective vaccine with worldwide immunisation coverage to restore normalcy to people's lives. As a result, lifelong protection from COVID-19 vaccinations is unlikely, and a regular immunization regimen might required in the future.

Traditionally, people from different origins and traditions used plant parts (flower, seed, leaves, and bark) and plant extract as an alternative medicine to treatdifferent ailments. These plants were used in folk medicine or traditional medicine. Various civilizations exist all across the world, thus different traditional medicines were developed to improve the quality of life. Some of these cultures introduced Indian traditional medicines, Chinese traditional medicines, Arabic traditional medicines.With the origin of Islam in 7th century, the knowledge about prevention of disease and cure was count in Arabian Peninsula. Medicinal opinions used in beginning of Islamic era are known as Tibb Al-Nabawi by Muslim society (Al-Rumkhani et al., 2016). Traditional Arabic or Islamic medicines (TAIM) originated hundreds of years ago and are still in use. Many traditional Prophetic medicinal plants have bioactive compounds and biological activities that could be used to develop and treat the COVID-19 symptomatically (Tahir ul Qamar et al., 2019).

In this review, systematic study of several TAIM plants with the biological activities (especially focusing on antiviral perspective) helpful to cure symptoms of COVID-19 has been dicussed. This study could pave the way for discovery and development of new antiviral medications to combat pandemic COVID-19.

## 2 Material and methods

This comprehensive study has been used to screenout some important plants, herbs, TAIM based isolates having efficacious properties to treat antiviral diseases, significantly COVID-19, identified through experiment and computational studies worldwide. These natural remides, their information, and their mechanism of action in human body has been studied. Data was collected from literature using different freely available resources, such as Google Scholar, SciFinder, SciDirect, PubMed, Scopus, and so on.

## 3 Results

Each plant’s multiple bioactivites have been discussed in detail involved in the treatment of antiviral infections thorugh different mechanism of actions (Table 1, Table 2, Table 3, Table 4, Table 5, Table 6).

**Table 1:** Pharmacological activities of bioactive compounds from *A. vera.*

Sl.No	**Biological Activity**	**Active Compounds**	**Influence on cell’s/disease biological mechanism**	**References**
1.	Antiviral	Lectins	Inhibits proliferation of CMC by interfering protein synthesis	(Wynn, 2005)
		Acemannan	Blocks reproduction of Herpes and AIDS virus, inhibits cell fusion and suppression through modification of glycosylation of virall infected cell.	(Li et al., 1993)
		Aloin	Phospholipid double layer destruction by integration into the viral envelope in VHS.	(Alves et al., 2004)
		Uronic acid	Protein synthesis inhibited by preventing aa-*t*-RNA association with the Ribosome.	(Rezazadeh et al., 2016)
		Ethanol	Inhibits autophagy that is induced by influenza virus in MDCK cells	(Choi et al., 2019)
		Aloe emodin	Cleavage of 3C like protease inhibit in SARS Corona virus	(MORIN, 2008)
		Chrysophanic acid	Blocks penetration of the virus into cell, initial cleavage of viral protein in polio virus	(MORIN, 2008)
		Zinc ionophores	stops replication of viruses	(te Velthuis et al., 2010)
2.	Immuno-modulatory	Acemannan	Increases nitric oxide and cytokine production in macrophages to increase the lymphocyte response to alloantigen (IL-1,6,IFN,TNF).	(Misir et al., 2014)
		Alprogen	Impedes the entry of calcium into mast cells to stop the synthesis of histamine and leukotrienes by mast cells	(Sahu et al., 2013)
		Lectins	Interaction can initiate signal transduction, create cytokines, and trigger effective immune responses against a variety of microbes.	(Hamman, 2008)
3.	Antiasthmatic	Protanoids	Shows effectiveness against chronic bronchial asthmatics.	(Koshak et al., 2018)
4.	Antipyretic	Saponin	Causes vasodilation and accelerates the decrease of phlebitis level.	(Guanche-Sicilia et al., 2021)
		Lignin	Reduce body temperature	(Rjasekaran et al., 2005)
5.	Antitussive	Polysaccharide HF1-Z	Polysaccharides that are taken orally and swallowed promote mucus and saliva production, which protects and covers cough receptors in the airways.	(Nosalova et al., 2006)

**Table 2:** Pharmacological activities of bioactive compounds from *O. europaea.*

Sl.No	**Biological Activity**	**Active Compounds**	**Influence on cell’s/disease biological mechanism**	**References**
1	Antiviral	Oleuropien	Inhibits the HIV-1 by binding with gp41	(Fredrickson, 2000)
		Calcium elonnate	Ability to penetrate and stop replication in infected cells. It can stop the development of reverse transcriptase and protease in retroviruses.	(Dhama et al., 2020)
		Cinnamic acid	Inhibits Integrase enzyme that is important for HIV replication	(Li et al., 1993)
		Acteoside	In HEp2 cells, there was a reduction in ERK activation, subsequent IFN-production, RSV multiplication, and virus-induced cell death.	(Song et al., 2016)
		Rutin	Inhibition of ACE2 receptors and decrease its binding ability with viral spike protein attachment	(Mittal et al., 2021)
		Apigenin	It is effective against EV71 infection because it prevents viral RNA from interacting with host transacting factors.	(Zhang et al., 2014)
		Hydroxytyrosol	HT binds with gp41 and cause a conformational change in the glycoprotein,and inhibit viral entry into target cells	(Lee et al., 2003)
2	Immuno-modulatory	Oleuropien	Induce lymphotes activation with morpholological characteristics that are similar to those of PHA-activated lymphocytes	(Schulte et al., 2007)
		Erthrodiol	Decreasing IL-1 β production,has strongest activity in reducing IL-6 production	(Marquez-Martin et al., 2006)
3	Antiasthmatic	Quercetin	Blocks IL-8 and MCP-1 production in airway epithelial cells by inhibiting signalling via the PI-3 kinase/AKT/NF-KB pathway.	(Nanua et al., 2006)
		Hydroxytyrosol	Pro-inflammatory cytokines including interleukins (ILs, tumour necrosis factor (TNF-), and chemokines like C-X-C motif chemokine 10 (CXC10) inhibition	(Richard et al., 2011)
		Rosmarinic acid	Inhibits inflammatory cell accumulation, production of Th2 cytokine, blocks phosphorylation of MAPK and NF-KB	(Caramori et al., 2008)
		Scopoletin	Treats bronchial illness	(Kooti et al., 2016)
4.	Antipyretic	Ethanolic leaf extract	Alleviate effects of pain and fever reactions initiators	(Alizadeh et al., 2014)
		Linoleic acid	Lowers the body temperature and cures fever	(Zhao et al., 2005)
		Caffeic acid	blocks the il1β and shows antipyretic activity	(Miles et al., 2005)
5.	Antitussive	Esculetin	Effective antitussive compound	(Ghanbari et al., 2012)
		Shikimic acid	Used to make Tamiflu drug that is effective against influenza virus A and B	(Saxena et al., 2012)

**Table 3:** Pharmacological activities of bioactive compounds from *T. foenum-graecum.*

**Sl. no.**	**Biological Activity**	**Active Compounds**	**Influence on cell’s/disease biological mechanism**	**References**
1.	Antiviral	Trigolleine	The major alkaloid, methyl betaine derivative	(Hasan Khan et al., 2019)
		Apigenin	Reduces viral IRES translational activity	(Patch et al., 2011)
		Orientin	Ability to associate the SARS-CoV-2 mutant model's overlapping amino acid residues	(Pastick et al., 2020)
		Diosgenin	Enhances expression of IFN- γ. This is significant since IFN-R/IFN-g combos have shown to be strongly anti-HCV	(Mehrafarin et al., 2010)
		Coumarin	SARS-CoV-2 inhibitor, which prevents the replicating polypeptide from being cleaved into several functional proteins	(Aanouz et al., 2021, Khan et al., 2021)
		Quercetin	Reduces the p24 level,gene expression of long (LTR), TNF- inhibition and IL-13 overexpression and inhibit viral infectivity	(Nair et al., 2002)
		Chlorogenic acid	Blocks the viral RNA synthesis in EV71-infected RD cells	(Chen et al., 2010)
		Luteolin	Hepatocyte nuclear factor 4 (HNF4) expression was inhibited in HepG2.2.15 cells to impede HBV replication, as well as HIV Tat protein function and HIV replication	(Bai et al., 2016, Mehla et al., 2011)
		Kaempferol	Inhibition of the 3a protein ion channel and counteracting virus production	(Zakaryan et al., 2017)
2.	Immuno-modulatory	Quercetin	*T*-helper cells are triggered to produce (Th-1) derived interferon (IFN-), and Th2-derived IL-4 is downregulated, when introduced to cultured blood mononuclear cells	(Nair et al., 2002)
3.	Antiasthmatic	Saponins	It blocks the release of phlogistic agents by inhibiting the pathways for the creation of inflammatory mediators like prostaglandins, histamine, secrotonin, and bradykinin	(Fanta, 2009)
		Chlorogenic acid	Reduced IL-6 and TNF- production, which is induced by influenza virus infection, which reduced lung tissue inflammation and damages.	(Wong et al., 2011)
		Luteolin	Inhibit mucus accumulation of airway through inhibition of GABAergic system	(Shen et al., 2016)
		Flavonoids	Protects the airways against oxidative stress and inhibits lipid peroxidation.	(Rahman et al., 2022)
4.	Antipyretic	Chlorogenic	Lowers the body temperature	(Chen et al., 2010)
5.	Antitussive	Saponin	Covers cough receptors	(Li et al., 2021b)

**Table 4:** Pharmacological activities of bioactive compounds from *F. carica.*

Sl.no.	**Biological Activity**	**Active Compounds**	**Influence on Cell’s/Disease Biological Mechanism**	**References**
1.	Antiviral	Quercetin	Effective against budding in MT2 cells that is produce by human *T*-lymphotropic virus	(Coelho-dos-Reis et al., 2011)
		Luteolin	Prohibits major protease of SARS-CoV-2, formsan alkyl and hydrogen bonds with Cys-145	(Dai et al., 2020)
2.	Immuno-modulatory	Ethanolic extract	Both cellular and humoral antibody responses are significantly improved	(Camero et al., 2014)
3.	Antiasthmatic	Quercetin	Reduce asthma-related diseases include eosinophil,neutrophil recruitment, bronchial epithelial cell stimulation, mucus and collagen secretion, and airway hyperactivity	(Odriozola-Serrano et al., 2008)
4.	Antipyretic	Ethanolic Extract	Reduce normal body temperature, has effect like common drug paracetamol	(Patil et al., 2010)
5.	Antitussive	Fruit Extract	Traditional usage	(Rahman et al., 2022)

**Table 5:** Pharmacological activities of bioactive compounds from *N. sativa.*

**Sl. no.**	**Biological Activity**	**Active Compounds**	**Influence on Cell’s/Disease Biological Mechanism**	**References**
1.	Antiviral	Nigellidine	SARS and COVID -19 viral inhibitors produced similar or better results than other effective medications.	(Salim and Noureddine, 2020)
		Thymohydroquinone	Reduction of docking energy by SARS-CoV-2 6LU7, endoribonuclease, ADP-ribos-1-phosphatase, RNA-dependent RNA polymerase	(da Silva et al., 2020)
2.	Immuno- modulatory	Thymoquinone	• Increase T.lymphocytes and NK cells immunological responses	(Salem, 2005)
			• Suppressed IL-2, IL-6, and PGE2 in T lymphocytes, as well as IL-6 AND PGE2 in monocytes, to effectively modulate asthma inflammation. • Inhibits othe formation of leukotrienes • It has antitussive properties due to its anti-inflammatory and brochodilatory actions, which are most likely mediated through opioid receptors.	(Koshak et al., 2018; (Srinivasan, 2018, Hosseinzadeh et al., 2008)
3.	Antiasthmatic	Nigellone	Histamine release suppression from mast cells	(Hajhashemi et al., 2004)
		α-Hederin	Lessens histamine and leukotriene release while raising PGE2 through mast cells to improve tracheal responsiveness	(Boskabady et al., 2011, Ikhsan et al., 2018, Saadat et al., 2015, Zhao et al., 2018)
		Volatile oil	Increase intratracheal pressure and respiratory rate	(Maurya et al., 2005)

**Table 6:** Pharmacological activities of bioactive compounds from *C. angustifolia.*

Sl.no**.**	**Biological Activity**	**Active Compounds**	**Influence on Cell’s/Disease Biological Mechanism**	**References**
1.	Antiviral	Kaempferol	Inhibits virus replication in brain of PRV-infected mice	(Zhao et al., 2018)
		Isorhamnetin	Blocks ACE2-spike protein interactions	(Yang et al., 2020)
		Emodin	Inhibits CK2, that is used by several viruses to phosphorylate proteins that are necessary for their survival	(Battistutta et al., 2000)
2.	Immuno-modulatory	Alkaloids, Flavonoids, Tannis & Phytosterols	Reduce the amount of reactive oxygen species production and protect immune cells.	(Jeong and Lachance, 2001)
		Vitamin E, & Iron	By boosting cellular components of hemopoiesis, it is important to growth and maturity of the immune system.	(Maggini et al., 2007)
		Polyunsaturated fatty acids	Important for neutrophil activity immune response	(Maggini et al., 2007)
		Zinc	Plays role in cytosolic superoxide dismutase activity, that helps neutrophils live longer by avoiding oxidation processes.	(Maggini et al., 2007)
		Alcoholic Extract	Stimulate serum protein synthesis, which is vital for the body's defence mechanisms	(Ilavarasan, 2001)
		Copper	Essential for enzyme cerruloplasmin that has function in humoral immunity	(Moyo et al., 2011)

### 3.1 Aloe vera

**Fig. 1:**
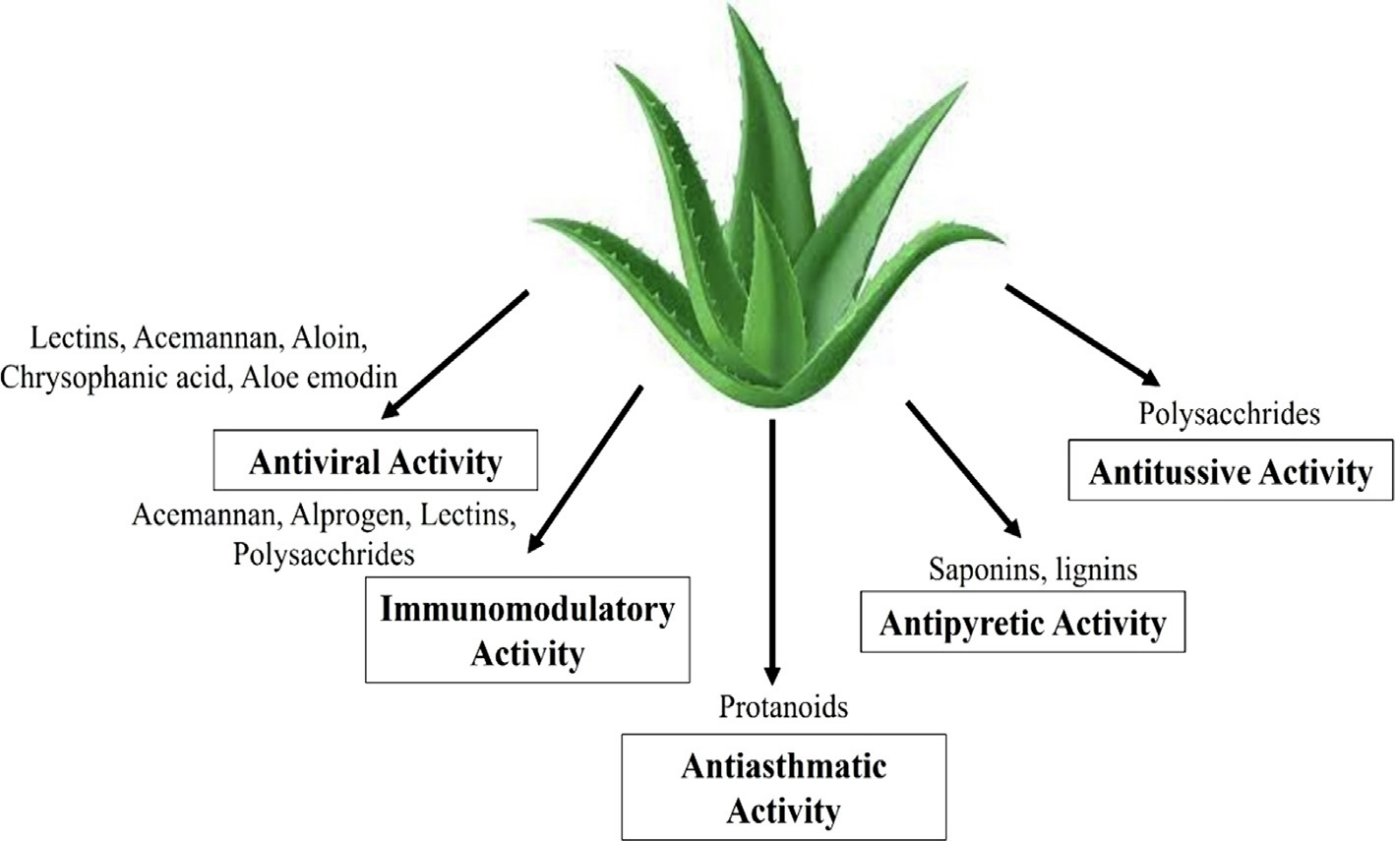
Pictorial representation of the plant *Aloe vera* with its bioactivities.

*Aloe vera L.* (*Aloe barbadensis Miller*) belongs to Aloaceae. Aloe word is originated from the Arabic term “Alloeh” or “Halal,” meaning “shiny sour material” and vera, is derived from the Latin wordtruth. This is a cactus-like perennial plant 60–100 cm tall with stemless or extremely short stemmed (Fig. 1) that grows in dry and hot climates (Joseph and Raj, 2010). The plant grows in Southern and Eastern Africa,as well as Mediterranean and other countries across the globe (Kumar et al., 2010, Yeh et al., 2003). Among diverse plant species *Aloe barbadensis* Miller, *Aloe chinesis* Bak, *Aloe indica* Royle, and *A. vera*. are some of the most oftenly used names of the plant. *A. vera* is considered a “healing” plant having medicinal value that has been used for more than 3000 years in different cultures (Mukherjee et al., 2014). It has anti-ageing, antiviral, antidiabetic and immunomodulatory properties (Wynn, 2005). According to a narration by Messenger of Allah (Peace Be Upon Him)*: “Do you know how much healing there is in the two bitter things: Aloe vera and cress?* (Bibi et al., 2021). Additionally, more than 200 active compounds including amino acids, saponins, anhraquinone, lignin, carbohydrates, enzymes, vitamins, minerals are found in the *A. vera* (Misir et al., 2014). Based upon its pharmacological importance this plant could be used to treat COVID-19 symptoms. The active compunds in *A. vera* with their influence on biological mechanism on disease prevention has been tabulated in Table 1.

#### 3.1.1 Antiviral activity

**Fig. 2:**
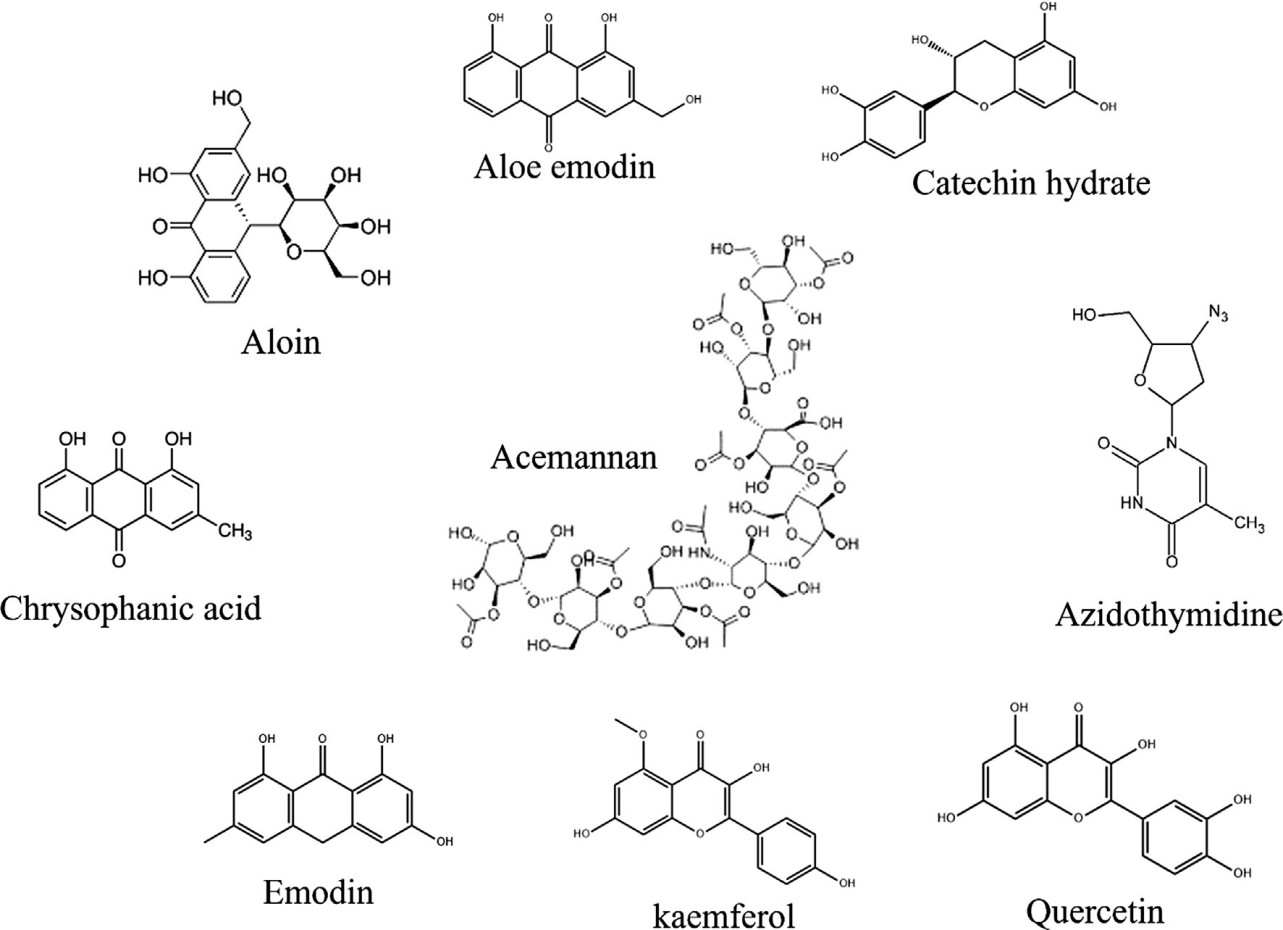
Chemical structures of active compounds from *A. vera.*

Considering *A. vera* as an effective antiviral agent, it contains lectins that is responsible to suppress the proliferation of Cytomegalovirus (CMV) growth in all cultures by interfering protein synthesis (Wynn, 2005). Moreover, the Acemannan (Fig. 2) found in *A. vera* is a key component of immune system that produces immune agents interferon and interlukin (Choi et al., 2007). Also, it may work in tandem with acyclovir (ACV), and azidothymidine (AZT) to prevent AIDS and Herpes virus reproduction (Mukherjee et al., 2014). Similarly, Acemannan shows antiviral activity against human immunodeficiency virus-1 (HIV-1) by modifying glycoprotein coats of viruses, glycosylation of virally infected cells thus inhibits the cell fusion and suppresses the virus release. Furthermore, aloin is an anthraquinone, also known as barbaloin. It has antiviral activity against Haemorrhagic Viral Rhobdavirus Septicaemia (VHS) anddamages the phospholipid bilayer layer thus integrates into the virus envelope (Alves et al., 2004). Furthermore, presence of polysaccharides (uronic acid), Anthraquinone and their derivaties (Tetracycline-analogous polyphenolic structures) hinder the protein synthesis by inhibiting the connection between aminoacyl-*t*-RNA and Ribosome. *A. vera* ethanol extract inhibits the apoptosis response in Madin-Darby canine kidney (MDCK) cells mediated by influenza virus (Choi et al., 2019).

It was observed that the hot crude extract of *A.vera* gel possesses an antiviral effects against Herpes Simplex Virus-2 (HSV-2) in the post attachment phases of virus replication (Zandi et al., 2007). Interestingly, upon inhibition of nucleic acid production and protein synthesis, aloe-emodin anthraquinone in aloe latex can render all viruses dormant, such as human α-herpes virus 3, influenza virus, and HSV Type I and Type II (Alves et al., 2004). Aloe-emodin shows antiviral action in case of SARS coronavirus by suppress the cleavage of 3C like protease, a viral replication enzyme that acts on the proteolytic process at the replication stage to aid viral replication (Mpiana et al., 2020). Emodin (Fig. 2) inhibits virus release by interfering with the 3a potein associated with ion channels of infected vero cells. This impact is crucial in the immunological response (Schwarz et al., 2011). Chrysophanic acid a natural anthraquinone shows antiviral effect against polio virus through preventing the viral entry into cell and initial cleavage of viral protein (Wynn, 2005). Tannins, saponins, flavonoids, and terpenoids were reported to have antiviral activity (Wani and Kumar, 2018). Instead of the surface tension of the extracellular medium, saponins demonstrate their activity by lysing the membranes of microorganisms (Nassiri Asl and Hosseinzadeh, 2008). Several individual compounds that have antiviral activity including quercetin, catechin hydrate, kaemferol, azidothymidine and acyclovir are also present in *A. vera* (Choi et al., 2019). Zn^2+^ hinder Arterivirus RNA polymerase and coronavirus action in vitro. In cell culture, zinc ionophores prevent these viruses from replicating (te Velthuis et al., 2010).

#### 3.1.2 Immunomodulatory activity

Scientific evidences suggest immunostimulatory and immunomodulatory properties in Aloe gel (Wong et al., 2011) due to the presence of polysaccharides in inner gel of *A. vera*. Similarly, Acemannan found in *A. vera*, is an immunostimulant partially purified carbohydrate mixture including 60 % acetylated mannan and other carbohydrates (e.g., pectins, hemicelluloses) that protects mice from UV-induced immune suppression (Bai et al., 2016). Acemannan boosts immunity via activating nitric oxide synthesis by macrophages and cytokines, as well as potentiating lymphocyte responses to alloantigen (IL-1, 6, IFN, TNF) (Li et al., 1993).

Calcium influx is inhibited by Alprogen into mast cells, preventing histamine and leukotriene production from mast cells driven by antigen–antibody interactions (Sahu et al., 2013). An additional research showed that lectins from *A. vera* gel have some immunomodulatory effects. The interaction of lectins with glycan moieties on the surface of immune cells causes these immunomodulatory effects. This connection activates signalling, produces cytokines, and induces effective immune responses against a variety of microbes (Hamman, 2008). *A. vera* extract (AVH200) reduces the cytokine secretions and activates the proliferation of healthy human blood T cells in vitro (Ahluwalia et al., 2013). The presence of Mannose rich polysaccharide components in *A. vera* gel were found to be responsible for enhanced production of antibodies in mice (Mukherjee et al., 2014).

#### 3.1.3 Anti-asthmatic activity

Protanoids, found in *A. vera* gel extract are the active compounds showed effectiviness against chronic bronchial asthmatics. If the patient has already been treated with steroid medications, the activity against asthma becomes in effective (Can et al., 2004).

#### 3.1.4 Antipyretic activity

Aloe vera saponin was found to cause vasodilation, that speeds up the reduction of venitis later eight hours of applying *A. vera* compress (Dwi Astuti et al., 2017). This further strengthened by another study conducted to examin phytochemical properties of saponin and how using it as a compress could help burn victims lower their body temperatures. Therefore, *A. vera* leaves were applied as a compress to cure fever (Surjushe et al., 2008). Furthermore, the lignin in *A. vera* aids in the prevention of fluid loss from the skin's surface (Rjasekaran et al., 2005).

#### 3.1.5 Antitussive

Studies showed that the substances with prominent peripheral mechanisms can lessen the frequency of coughing while having little influence on its amplitude during expiration. Cough frequency is determined by the state of cough receptors. The HF1-Z polysaccharide effect on IA^-^, IA^+^ and NE (norepinephrine) showed the mechanism of cough suppression. The cough suppression properties of *A. vera* is considered due to the preventative reaction of polysaccharides on cough receptors. Even if they do not come to the airduct when eaten, polysaccharides protect and cover cough receptors in airduct as well as extra-respiratory functional structures that trigger coughing (Nosalova et al., 2006).

### 3.2 Olea europaea

**Fig. 3:**
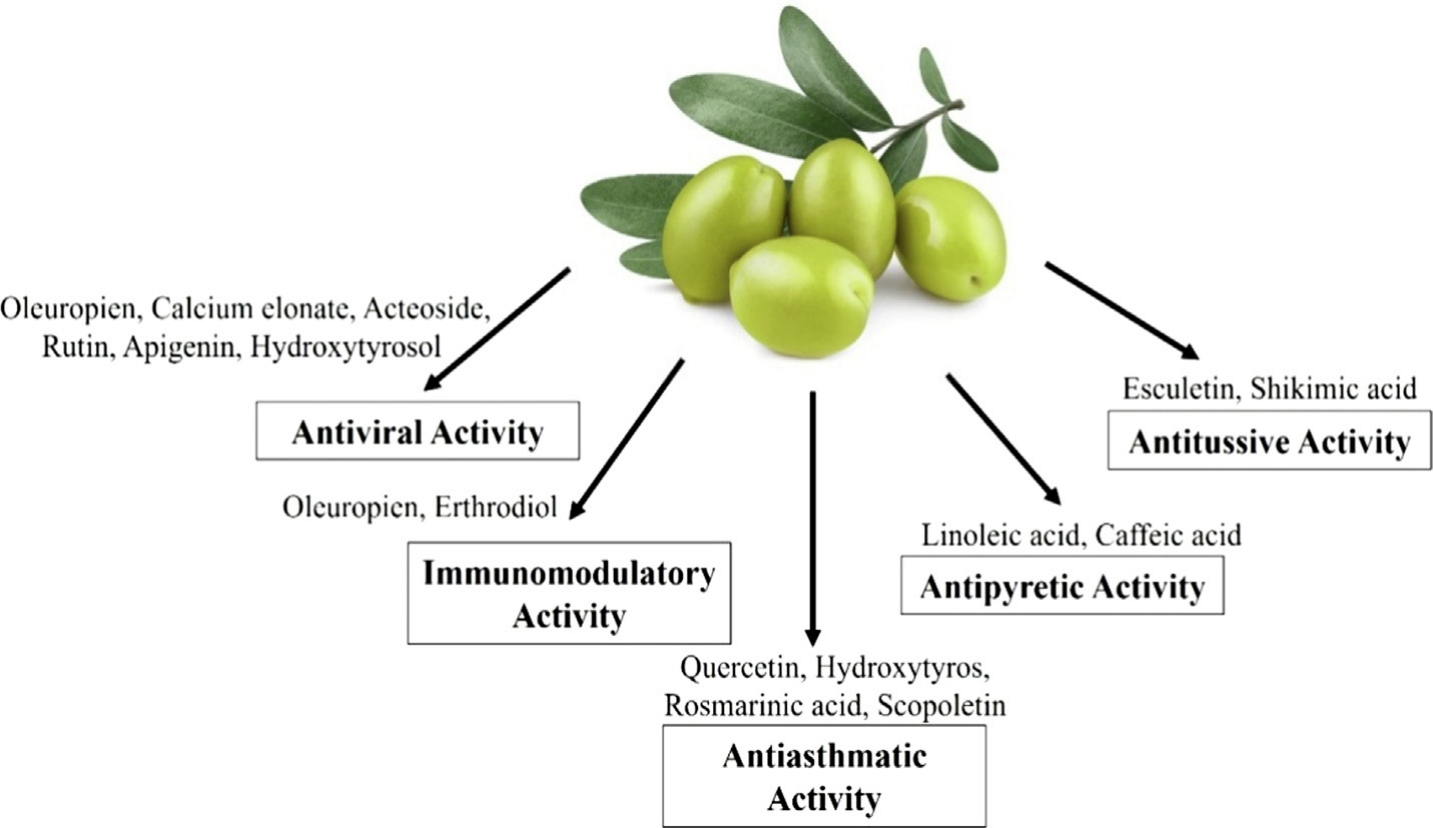
*Olea europeae* with its active compounds.

The family Oleacease includes *Olea europaea*. The genus Olea is named after Greek word “elaia” and Latin word “oleum” in spite of that it has approximately eighty other names (Médail et al., 2001). It is a short evergreen tree, approcximately 12–21 feet high with the upright blooms racemes and with lanceolate small and axillary leaves (Ghanbari et al., 2012) (Fig. 3). This plant is spread in northern Iran along Caspian Sea's southern end, as well as along the coasts of southeastern Europe, northern Africa, and Western Asia (Gouvinhas et al., 2017). The Oleaceae family thrives in Malaysia and Asians tropical and temperate zones (Pérez et al., 2005). It is a holy plant for Muslims and Jews, mentioned in their holy scripts as a wonderful tree and fruit. According to a narration stated by Muslim’s Prophet (PBUH)” it (*O. europaea*) is a blessed tree, you can eat the oil and use it”.

*O. europaea* common name Olives, due to their bitter taste are not consumed as a natural fruit instead they are crused for oil production (Kanakis et al., 2013). Moreover, specific parts of the *O. europaea* plant are utilized in traditional medicine around the world (Al-Aboudi and Afifi, 2011). From its numerous portions, secondary metabolites such as flavone glycosides, flavanones, iridoids, iridane glycosides, biophenols, triterpenes, derivatives of benzoic acid, xylitol, sterols, isochromans, sugars, and a few more kinds are isolated. Flavonoids, secoiridoids, and secoiridoid glycosidesare extremely ubiquitous in every sections of *O. europaea* (Jerman et al., 2010). Experimental evidences indicated that presence of phenolic compounds in *O. europaea* have crucial importance in lowering the risk and severity of the disease (Abaza et al., 2015). Thus *O. europaea* may serve as a possible pharmacological plant for the management of COVID-19 symptoms.

#### 3.2.1 Antiviral activity

Varios studies reported antiviral activities of *O.europaea*. One of the significant compounds oleuropien is considered to have antiviral potential against hepatitis, mononucleosis, and herpes. Furthermore, oleuropien prevents HIV from entering healthy cells by inhibiting the surface glycoprotein (gp) subunit HIV-1 gp-41 (Fredrickson, 2000). Cinnamic acid extract from olive is another compound showed antiviral activity (Abbattista et al., 2021) against HIV. It inhibits the HIV-1 integrase enzyme responsible for virus replication (Yadav et al., 2019). Similarly, elenolic acid of calcium salt extractwas found to be a wide range antiviral factor that was effective against every viruses tested (Tripoli et al., 2005). Several viruses such as Rhinovirus, Myxoviruses, HSV- I, Shingles, Polio 1,2,3, different strains of influenza and *para*-influenza viruses are inhibited by calcium elenolate a derivative of elenolic acid (Li et al., 2021a). The amino acid synthesis is inhibited by calcium elenote by interfaring virus molting, fledgling, or aggregation at plasma membrane.

**Fig. 4:**
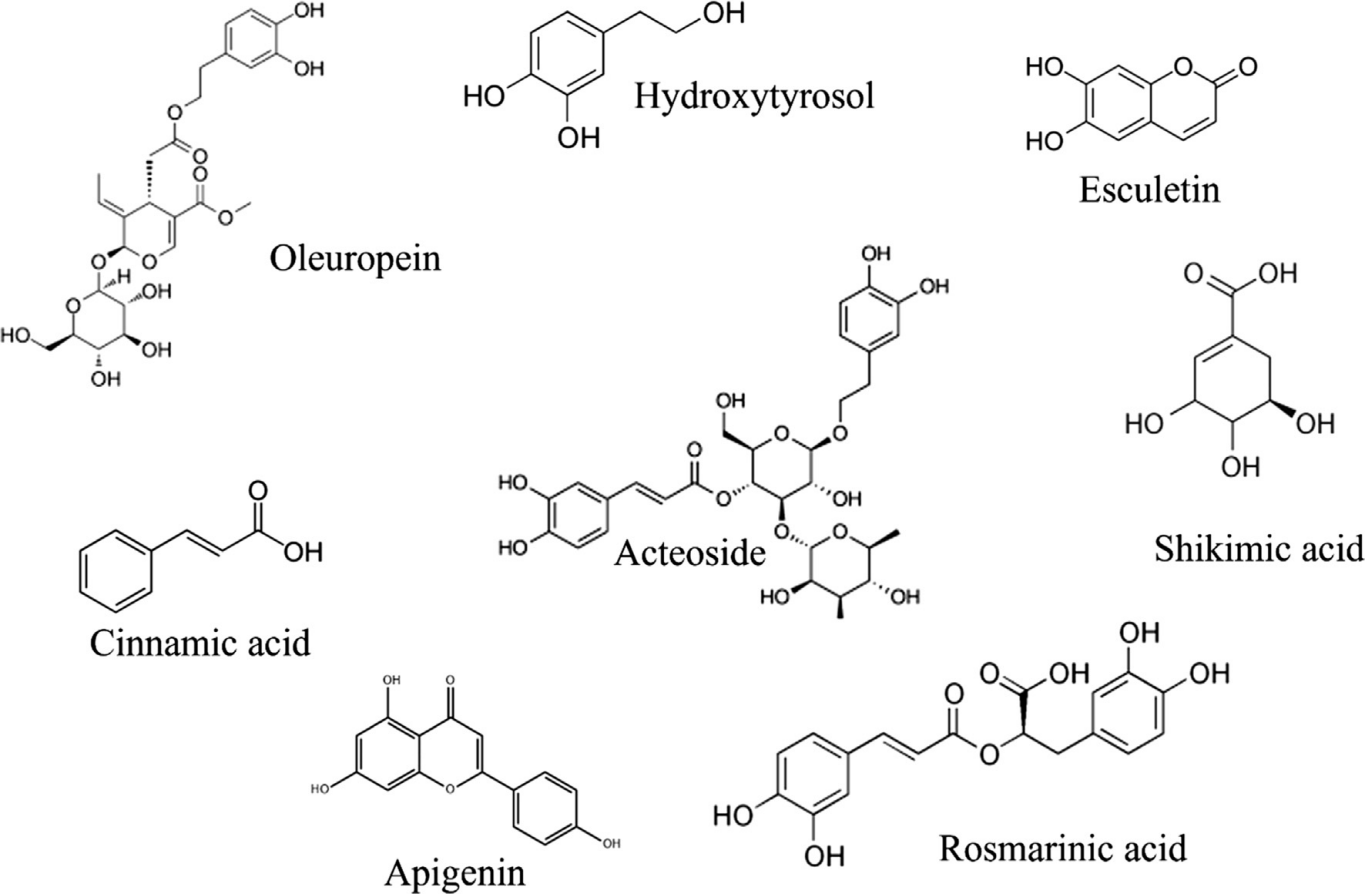
Active constituents from *O. europaea.*

Through the activation of (Extracellular signal-regulated kinase) ERK and IFN-γ (interferon gamma) production, acteoside has antiviral efficacy against vesicular stomatitis virus (VSV) and influenza virus. Respiratory Syncytial Virus (RSV) replication and viral-induced cell death in HEp2 cells were likewise reduced by acteoside (Zhao et al., 2018). Rutin and apigenin (Fig. 4) are antiviral flavonoids extracted from fruit and pulp of olives. Previous studies reported that Angiotensin Converting Enzyme-2 (ACE2) help SARS-CoV-2 to enter into target (Anand et al., 2003). Inhibiting the ACE2 receptor may lessens the power to bind to virus S protein attachment. Rutin binds to the ACE2 receptor via amino acid residues (PHE4, LEU-29, GLN-388, PRO-389, LEU-391 ASP-30, ASN-33, VAL-93, ALA-99, LEU-100, ALA-387, ASP-350, ALA-387, and ARG-393) with a high affinity (Mittal et al., 2021). However, ASP-30 is considered vital role in receptor-binding domain (RBD) binding (Gupta et al., 2021).

Additionally, viral RNA reaction to the host transactivation element in EV71 infection (enterovirus 71) (Zhang et al., 2014) was stoped by Apigenin from olive fruit as reported by Zhang. In another study, olive leaf extract was observed to have a potent anti-HIV-1 activity due to the presence of hydroxytyrosol (a phenolic alcohol having antimicrobial and neuroprotective effects) compound mixture that prevents severe infections, HIV-1 cell-to-cell transfer, and viral multiplication in infected cells. Moreover, the leaf extract contains the HIV-1 inhibitors, Molecular analysis showed that Ole and HT bind to the hydrophobic region on the surface of the HIV-1 envelope glycoprotein. Moreover, HT was found to be the primary ingredient for binding to viral gp41. Upon binding, a conformational shift has been introduced that may prevent viral entrance into target cells (Liu et al., 2018).

#### 3.2.2 Anti-asthmatic activity

Traditionally, to cure bronchial asthma, a warm water soup of the dried plant is taken orally (Sakipova et al., 2020). Quercetin reported, cause bronchodilation within living and artificial environment. Oliveria et al. 2015 investigated quercetin effect on smooth muscle contraction and cytokine levels, ex-vivo as well as its curative ability on a mouse model of asthma. The study revealed quercetin can reduce the creation of inflammatory cytokines production, tranquility of tracheal, decrease entire range of cells in BALF (bronchoalveolar lavage fluid) and EPX in remedy of lungs (Oliveira et al., 2015). Quercetin has the capacity to diminish airway hyper reactivity, which is a key feature of allergic asthma. It inhibits Interleukin-8 (IL-8) and MCP-1 production in airway epithelial cells by inhibiting signalling via the PI-3 kinase/AKT/NF-KB pathway (Nanua et al., 2006).

Hydroxytyrosol exhibits antibacterial activity against respiratory tract pathogenic pathogens (Odriozola-Serrano et al., 2008) was found an active compound in olives. Hydroxytyrosol has strong anti-inflammatory effects, that includes Nitric Oxide and prostaglandin E2 inhibition, reduces the release of pro-inflammatory cytokines (IL)-1α, IL-1β, IL-6, IL-12, Tumor Necrosis Factor-α (TNF-α), chemokines like (CXC10))/interferon γ-induced protein 10, (C—C motif) ligand 2 (CCL2)/(MPC-1) and reduces gene expression of inferential NO synthase (iNOS), IL-1α, CXCL10/IP-10, macrophage inflammatory protein-1β (MIP-1β), matrix metalloproteinase-9 (Richard et al., 2011).

Similarly, Rosmarinic acid (RA) anti-asthmatic compound extract from olive pomace (Abbattista et al., 2021). The generation of Th2 cytokines, the phosphorylation of MAPK and NF-KB, the accumulation of inflammatory cells, and the mRNA expression of inflammatory genes were all suppressed by RA (Caramori et al., 2008). Scopoletin compound which is used to treat bronchial illness and asthma present in the bark of *O. europae* (Kooti et al., 2016).

#### 3.2.3 Antipyretic activity

Olive tree and it’s extract has long been associated with good health, and is also used in traditional medicine to treat conditions like fever (Gouvinhas et al., 2017). Ethanolic extract of olive leaf plays a key functional role in alleviation of the negative effects of ache and pyrexia (Alizadeh et al., 2014). Oleuropien belongs to the secoiridoids showed antipyretic effects (Gouvinhas et al., 2017). Caffeic acid and oleuropein present in olive leaves, inhibit the il1β and have antipyretic activity (Miles et al., 2005).

#### 3.2.4 Immunomodulatory activity

Olive leaf extracts and oleuropein has a immunostimulatory effects on normal lymphocytes. They are capable of inducing lymphocytes activation with morpholological characteristics that are similar to those of PHA-activated lymphocytes (Schulte et al., 2007). Pentacyclic triterpenoid including Erythrodiol and Uvaol compound present in fruit of various kinds of *O. europaea* were studied for immunomodulatory action on human mononuclear cell lymphokine creation. Erythrodiol is the most effective inhibitor of IL-1 production. This compound is considered the most effective lowering IL-6 production (Marquez-Martin et al., 2006).

#### 3.2.5 Antitussive

Antitussive compound Esculetin a hydroxycoumarin found in bark of Olive (Ahmad et al., 2013, Mehrafarin et al., 2010). Shikimic acid, a compound found in *O. europaea* is used to made anti-influenza drug oseltamivir. Oseltamivir is marketed as product of Tamiflu. It is use to cure symptoms caused by influenza virus. Tamiflu is a neuraminidase inhibitor that act as an effective compound against swine flu (Saxena et al., 2012). The bioactivity of the important compounds found in olive and their mechanism of action in disease condition has been tabulated (Table 2).

### 3.3 Trigonella foenum-graecum

**Fig. 5:**
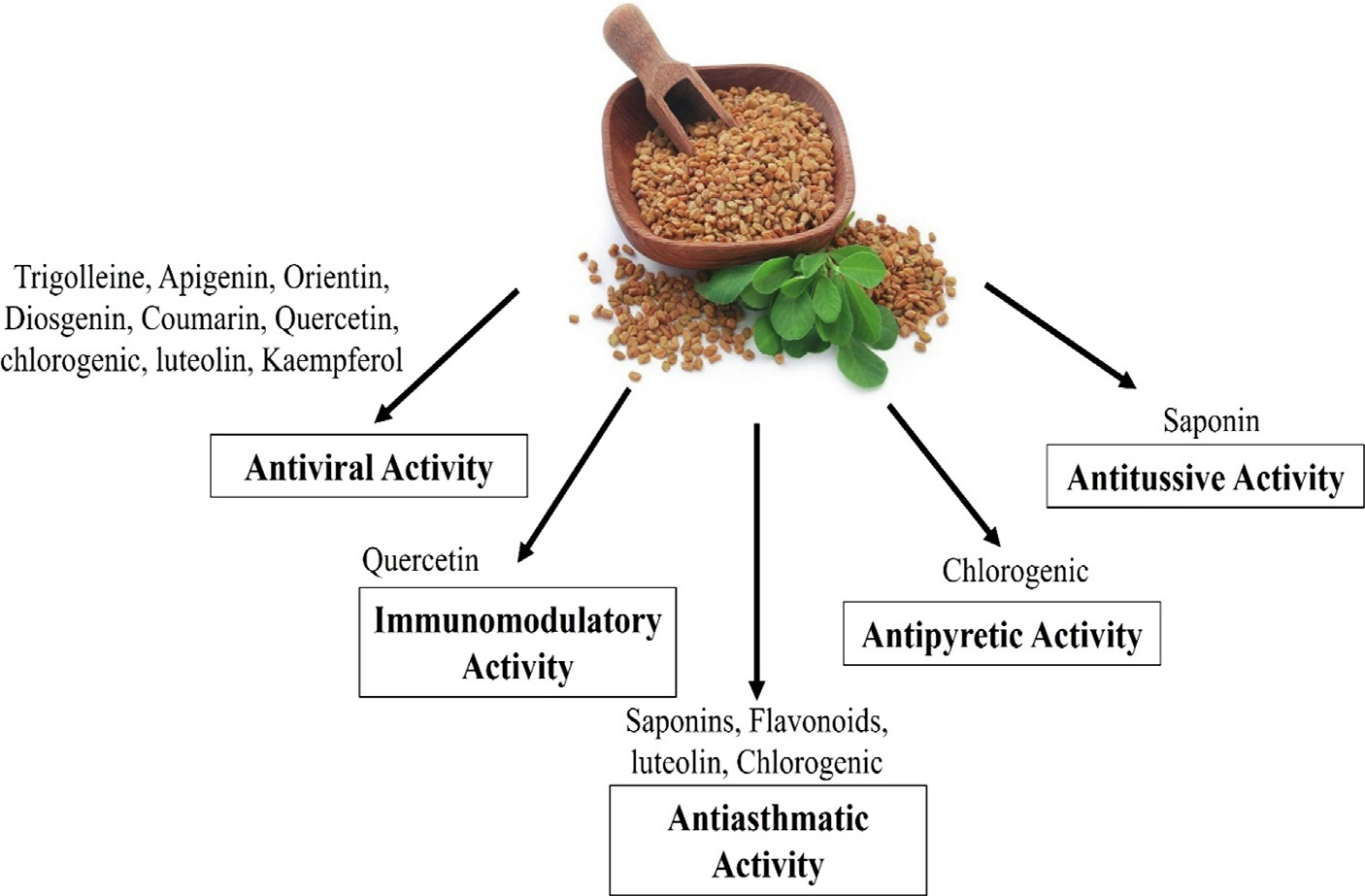
Schematic representation of the active compounds from *T. foenum-graecum* with associated bioactivities.

*Trigonella foenum-graecum* plant relates to order Rosaceae, Legouminosae family, Papilonaceae subfamily and trifolia genus *Trigonella* L. (Bibi et al., 2020). It is commonly known as Fenugreek. The term is derived from Latin word foenum-graecum, which means hay in Greek, because plant was formerly used to make substandard hay (Flammang et al., 2004). Trigonella gets its name from an antique Greek word that means “three-angled” (Wani and Kumar, 2018) (Fig. 5). Probably related to trigonal shape of theits bloom. Fenugreek is used as herb (leaves) as well as a spice (seed) in many countries worldwide as well as medicinal plant (Parthasarathy et al., 2008). China, India, Turkey, Canada, Australia, Africa, and Southern Europe are the largest producers of fenugreek (Rahman et al., 2022).

**Fig. 6:**
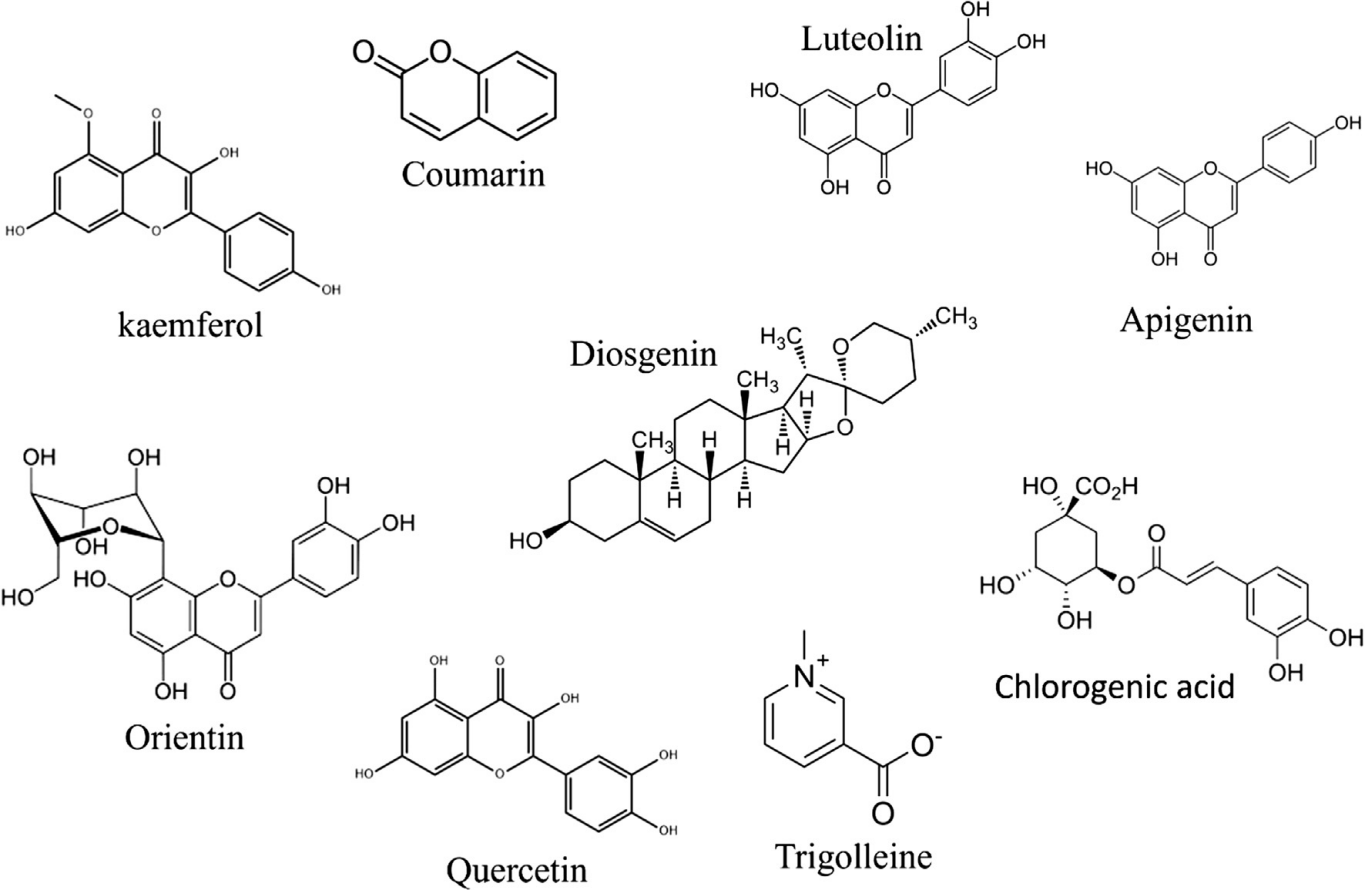
Chemical structures of compounds from *T. foenum-graecum.*

Fenugreek is a source of minerals, protein and vitamin (Parthasarathy et al., 2008). Because of its medical benefits, it is is widely grown throughout the world (Mehrafarin et al., 2010). It’s seeds carry 45–60 % carbs, 20–30 % proteins, 5–10 % fats, C_5_H_5_N, alkaloids cheifly trigonelline (0.2–0.38 %), choline, gentianine and carpaine, flavonoids, apigenin **(**Fig. 6), luteolin, orientin, and saponins (0.6–1.7 %) (Mehrafarin et al., 2010). Traditionally in Muslim’s culture, it is being used to treat influenza, head colds, bronchial asthma, pneumonia, sore throat tuberculosis, hay fever and sinusitis (“Fenugreek: An Herb with Impressive Health Benefits,” n.d.). According to the narration to seek cure in Hulba (Methi) (Khan, n.d.; Muhammed and Shamsi, 2016).

Trigonelline (Fig. 6) is prime metabolic substance present in this plant. It has potential to treate diabetes mellitus and drop cholesterol level in blood. It is also used for cancer and migraine. According to experts of folk medicine, fenugreek possess warm as well as dry nature and its leaflets are used in treatment of cough, and back pain. Additionally, fenugreek and honey mixture is used traditionally to treat asthma and inner edemas (Basch et al., 2003). Based on the above mentioned activities and compounds from fenugreek, it is tempting to propose that it might serve as a SARS-CoV-2 therapeutic agent.

#### 3.3.1 Antiviral activity

Antiviral compound Trigonelline is a by-product of nicotinic acid and prime alkaloid present in the fenugreek seeds (Hasan Khan et al., 2019). The major antiviral flavonoid found in this plant includes apigenin and orientin. Apigenin showed antiviral activity against Foot and Mouth Disease (FMD) virus. Viral Internal Ribosome Entry Sites (IRES) driven translational activity is inhibited by apigenin. The IRES region in the FMDV genome's 5′-UTR begins cap-independent viral genome translation (Patch et al., 2011). Similarly, Orientin can bind to the overlapping amino acid in the SARS-CoV-2 spike model which is considered liable for receptor GRP78 binding. Inhibition of the COVID-19 mutant protein interactions with the host receptor GRP78 is expected to lessens the viral infection rate (Pastick et al., 2020).

Diosgenin was revealed to be an interesting bioactive compound in some viral diseases. Current researches suggest that taking diosgenin can modulate some aspects of acquired immunity and increase IFN-g expression. This is significant since IFN-R/IFN-combos have shown to be strongly anti-HCV (Ivashkiv, 2018). Phenolic compound coumarin is similar in structure to protease and might be the inhibitor against the SARS-CoV 2 (Aanouz et al., 2021). Protease (also known as peptidase) is a thiol, that is required for virus replication by cleaving the replicating polypeptide into several functional proteins (Khan et al., 2021). Additionally, quercetin showed antiviral activity against HIV as it inhibits key enzymes such as reverse transcriptase, integrase, and protease (Li et al., 2014). Quercetin reduces p24 level, long terminal repeat (LTR) gene expression, and viral infectivity when given to HIV-infected peripheral blood mononuclear cells (PBMNc) and compared to HIV-infected controls through suppressing TNF- and upregulating IL-13 (Oliveira et al., 2015).

Phenolic bioactive compound such as chlorogenic acid shows antiviral activity due to it is capasity to inhibit the RNA synthesis in Enterovirus-71 (EV71) infected Rhabdomyosarcoma (RD) cells at initial stage (Chen et al., 2010). Luteolin is an antiviral substance that works against EV71, Hepatitis B Virus (HBV), and HIV via several methods. It inhibits the Hepatocyte nuclear factor 4 (HNF4) expression to stop multiplication of HBV in HepG2cells (Bai et al., 2016). It also disables the HIV Tat protein's ability to prevent HIV replication (Mehla et al., 2011). Kaempferol glycoside shows antiviral activity through inhibition of the 3a protein ion channel and counteracting virus production. As a result, additional stages of the viral life cycle can be disrupted (Zakaryan et al., 2017).

#### 3.3.2 Anti-asthmatic activity

Some of the antiasthmatic bioactive compounds present in several plant parts of fenugreek including inflammatory mediators such as histamine, leukotrienes, tryptase and acetylcholine cause bronchoconstriction in the early stage of asthma (Choi et al., 2007). The plant is used to treat asthma as it contains various phytoconstituents. These phytoconstituents showed different biological activities and bronchodilation activity. The steroidal saponins found in the plant, blocks the production routes of inflammatory mediators such as prostaglandins, histamine, secrotonin, and bradykinin.Various evidences suggest that the steroidal saponins hinder lipooxygenase and/or cyclooxygenase pathway (Caramori et al., 2008). Chlorogenic (CHA) may lessens the inflammation and lung tissue damage caused by interleukin 6 and TNF-a production during influenza virus infection (Wong et al., 2011). Luteolin flavonoid inhibits airway mucus accumulation by inhibiting the GABA ergic system (Shen et al., 2016). Flavonoids are a type of polyphenol with a low molecular weight that can help with asthma treatment. Flavonoids may suppress lipid peroxidation and defend lungs from oxidative stress (Hussain et al., 2018).

#### 3.3.3 Antipyretic activity

Fenugreek tea is used to perspire, eliminate toxicity and reduce fever period. In addition to the preceding activity the fenugreek leaves also exhibited antipyretic activity. Antipyretic alkaloid bioactive compound Gentianine is present in fenugreek seed (Yoshinari and Igarashi, 2010). Phenolic compound chlorogenic also has antipyretic activity (Chen et al., 2010).

#### 3.3.4 Immunomodulatory activity

Previously, it was revealed that herbal extract of fenugreek has immunostimulatory properties. *T. foenum-graecum* extract significantly increases the delayed type of hypersensitive reactions, macrophage phagocytic capability and phagocytic index, as well as lymphocyte proliferation in mice. These findings indicate the immunostimulatory effect of fenugreek in the prevention of a many of diseases (Bin-Hafeez et al., 2003). Moreover, querectin has been reported to possess immunomodulatory property. When administered in cultured blood peripheral mononuclear cells, it upregulates Th2-derived IL-4132 and induces *T*-helper cells to create (Th-1) derived interferons (IFN).

#### 3.3.5 Antitussive

Saponins in fenugreek plant plays a vital role in cough-suppression. Its pharmacokinetics and structural makeup are comparable to those of plant’s polysaccharides. Although saponin cannot be taken orally, it is instinctively associated with the vagal neurons of the stomach. It also encourages the production of mucus in the lower airways, which shields the cough receptors (Li et al., 2021b), hence prevent cough. The active compounds found in fenugreek plant and their role in diseased condition has been summerised (Table 3).

### 3.4 Ficus carica

**Fig. 7:**
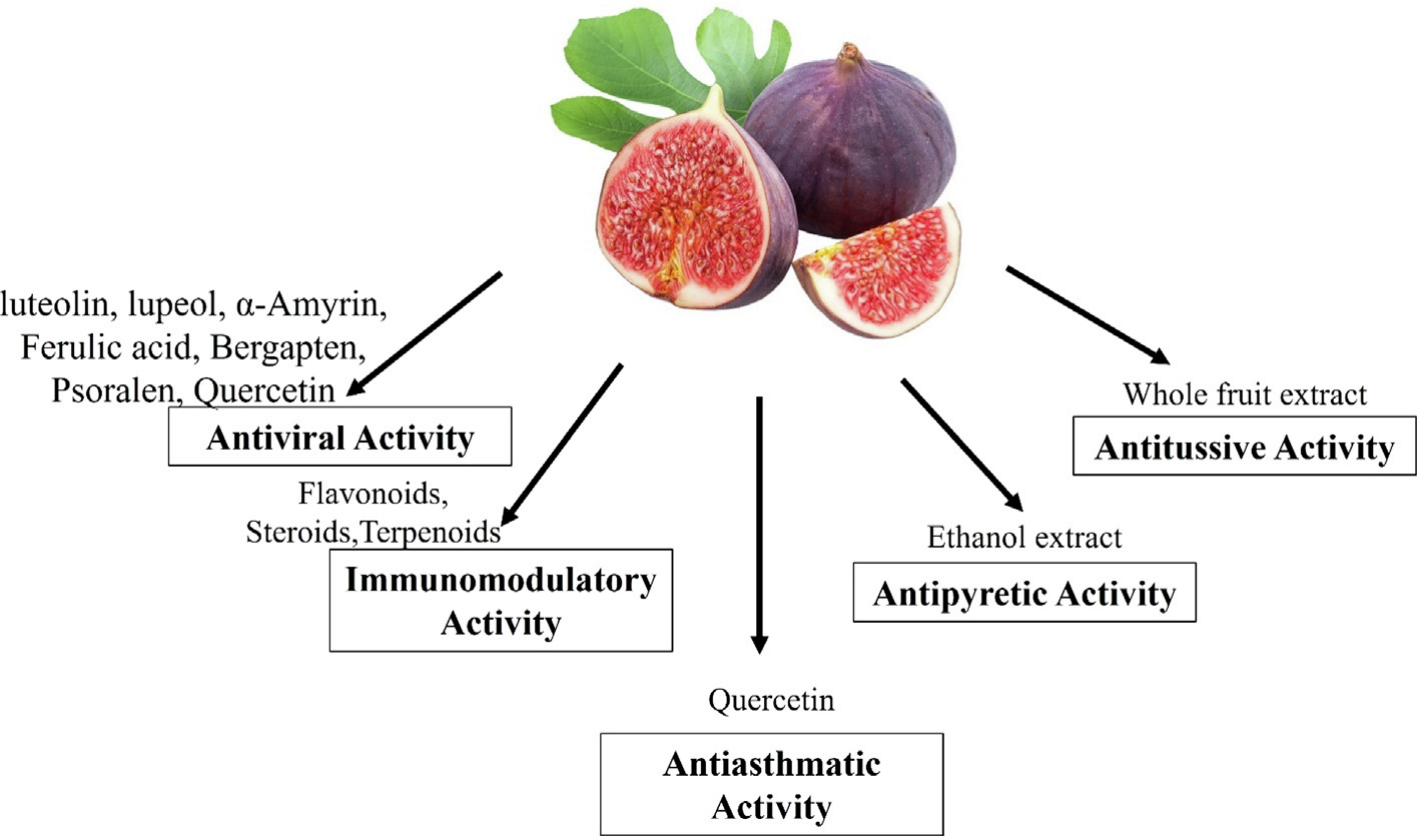
Diagramatical representation of the plant *F. carica* with its active compounds and associated bioactivies.

*Ficus carica Linn*. is generally known as “Fig”. *Ficus carica* is a member of Urticales order and Moraceae family (Barolo et al., 2014). The fig tree has many spreading branches, a trunk that is 7 feet in diameter, and a height of 20 feet^150^. Barks are grayish and roots are not adventitious. The leaves are thin and stemmed, with leaves on the palm, heart-shaped base, wavy or irregular dentate edges, blunt-tipped, rough, hairy surface (Fig. 7). Figure is known to be one of the first plants cultivated by human and oldest known fruit tree (Barolo et al., 2014). It isbeing cultivated worldwide and grows in dry warm climates. This plant is grown commercially in United States and Chile, as well as in Arabia, Persia, China, India, and Japan (Kumar, 2021). It has a religious importance in Muslims and Christanity (Slavin, 2006). According to a narration:“Eat fig; it is helpful for rheumatism and relieves piles” (Refaat et al., 2020).

**Fig. 8:**
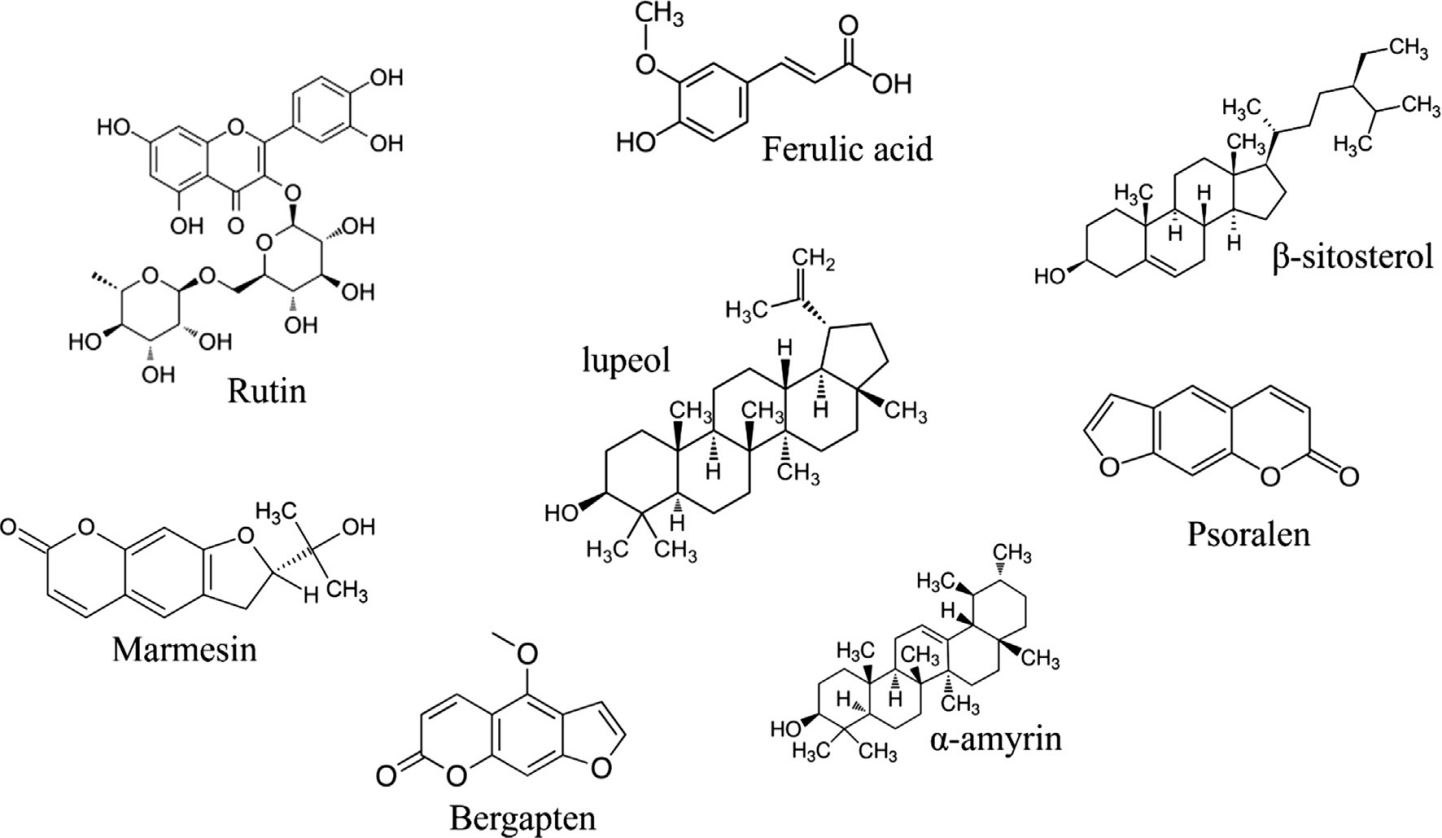
Chemical structures of active compounds from *F. carica.*

Furthermore, different parts of the plant are utilized traditionally to cure various ailments. Gastrointestinal, respiratory, inflammatory and cardic disorders are more noticeable diseases (Parthasarathy et al., 2008, Patil et al., 2010). Moreover, some important vitamins, minerals, organic acids, carbohydrates, sugars, and phenolic compounds are isolated from the dried fruit of fig (Jeong and Lachance, 2001, Slatnar et al., 2011, Veberic et al., 2008). Phytochemicals including sterols, coumarins, flavonoids, triterpenoids, anthocyanins etc., found in various parts of the plant. Leaves contain rutin, bergapten, marmesin (Fig. 8), stigmasterol, lupeol, β-sitosterol, ficusogenin, and tyrosine moisture, protein, fat, crude fiber, ash, pentosans, and carotene. Dried seeds of figs has fixed oil including the oleic acid, linoleic acid, palmitic acid linolenic acid,stearic acid, arachidic acid (Chang et al., 2005, Li et al., 2006, Saeed and Sabir, 2002, Wu et al., 2002). 6-O-linoleyl-β-d-glucosyl-β-sitosterol, resin, caoutchouc, albumin, sugar, cerin, malic acid, rennin, proteolytic enzymes, diastase, esterase, and peroxidase are isolated from latex (Rubnov et al., 2001). Cyanidin-3-O-glucoside, cyanidin-3-Orhamnoglucoside, saturated fat, cholesterol, salt, insoluble carbohydrates, protein, vit A, vit C, Ca, Ag are all found in fruit. Roots contain psoralen and bergapten (Li et al., 2006, Wu et al., 2002). These compounds from fig could be used to treat COVID-19 symptomatically (Islam et al., 2021).

#### 3.4.1 Antiviral activity

The antiviral activity of the fig was examined by Houda *et al.* in five different extracts (methanol, ethyl ethanoate, hexane–ethyl acetate (v/v), and chloroform) of the air-dried fruits in artificial medium, against (HSV-1) herpes simplex type-1, (ECV-11), and adenovirus type-11. The study revealed that the hexanic and hexane–ethyl acetate extracts suppress virus proliferation.The antiviral assays were performed by the parameters such as inhibition of adsorption, invasion, intracellular prohibition and virucidal potential.The antiviral potential was determined using the results of cytopathogenic effects. On vero cells, none of the extracts mentioned had any cytotoxic impact. It was concluded that *F. carica* fruits has the antiviral activity due to its main active phytocontituents, such as ferulic acid, 5-O-caffeoylquinic acids, coumarins including bergapten and psoralen (Fig. 8).

Moreover, this plant has high therapeutic values and also known for its elevated confrontation to stress conditions. Thus, the phytochemical compounds present in Ficus fruits can be an effective therapeutic compound against COVID-19 infections (Lazreg Aref et al., 2011). *F. carica* latex found to be responsible to reduce titres of virus, produced through CpHV-1-infected MDBK cells. The latex help to hinder the replication of CpHV-1 (Camero et al., 2014, Islam et al., 2021, Rani et al., 2022). Quercetin has also been discovered to have antiviral properties. Quercetin was found to be effective against human *T*-lymphotropic virus-induced budding in MT2 cells (Coelho-dos-Reis et al., 2011, Dey et al., 2022). Quercetin showed activity against dengue virus type-2 (Mir et al., 2016).

Due to their high binding affinities, the top three F-latex substances evaluated, luteolin, lupeol, and α-amyrin, may be used as SARS-CoV-2 main protease inhibitors. Lupeol (-12.5 kcal/mol), α-amyrin (-7.9 kcal/mol), and luteolin (-7.4 kcal/mol) had higher binding affinities than the ketoamide (-7.3 kcal/mol), according to a molecular docking investigations. Analysis revealed that lupeol creates non-covalent alkyl bonds with the essential catalytic residues His-41 and Cys-145, as α-amyrin does. With Cys-145, luteolin forms an alkyl and hydrogen bond. As a result, it may inhibit the primary protease of SARS-CoV-2. With an additional binding site residues, these three substances form a potent non-covalent bond.These results are corroborated by contemporary studies that used inhibitor compounds to form covalent and non-covalent interactions with the His 41, Met 49, Tyr 54, Phe 140, His 164, Met 165, Glu 166, Pro168, Asp 187, Arg 188, and Gln189, His 164, Met 165, Glu 166, Pro168, Asp 187 residues (Dai et al., 2020, Jin et al., 2020).

#### 3.4.2 Anti-asthmatic activity

It was observed that in comparison to conventional asthmatic drugs and steroid inhalers that reduce mucus secretion, quercetin isolated from fig has significant antiasthmatic effect. In addition, bronchial epithelial cell stimulant,eosinophil, neutrophil enrollment, mucus and collagen synthesis, and airway hyperactivity have all been linked to quercetin. Clinical trials have demonstrated that quercetin can be used to prevent or treat asthma in humans (Odriozola-Serrano et al., 2008).

#### 3.4.3 Antipyretic activity

According to Patil Vikas et al., 2010 ethanolic extract of figs leaf has a substantial antipyretic reaction in yeast-induced body temperature elevation in rats, and its impact is comparable to that of paracetamol used as a common medicine. *F. carica* extract also helps to lower normal body temperature (Patil et al., 2010).

#### 3.4.4 Immunomodulatory activity

Due to the numerous pharmacological actions, such as antioxidant and immunomodulatory properties, herbal phytochemicals, including flavonoids, steroids, etc., have drawn a lot of attention recently (Koshak et al., 2018, Salem, 2005). Patil et al. 2010 investigated the immunostimulatory effects of an ethanolic extract of Fig. Different haematological and serological assays in mice were used in the study. Extract administration significantly boosts both cellular and humoral antibody responses (Chen et al., 2020) showing the immune modulatory properties of fig.

#### 3.4.5 Antitussive activity

In Persian traditional medicine, fig fruit extract is being used to treat coughs, sore throats, and as an expectorant (Al-Rumkhani et al., 2016). Different compounds of pharmacological importance has been summerised in Table 4.

### 3.5 Nigella sativa

**Fig. 9:**
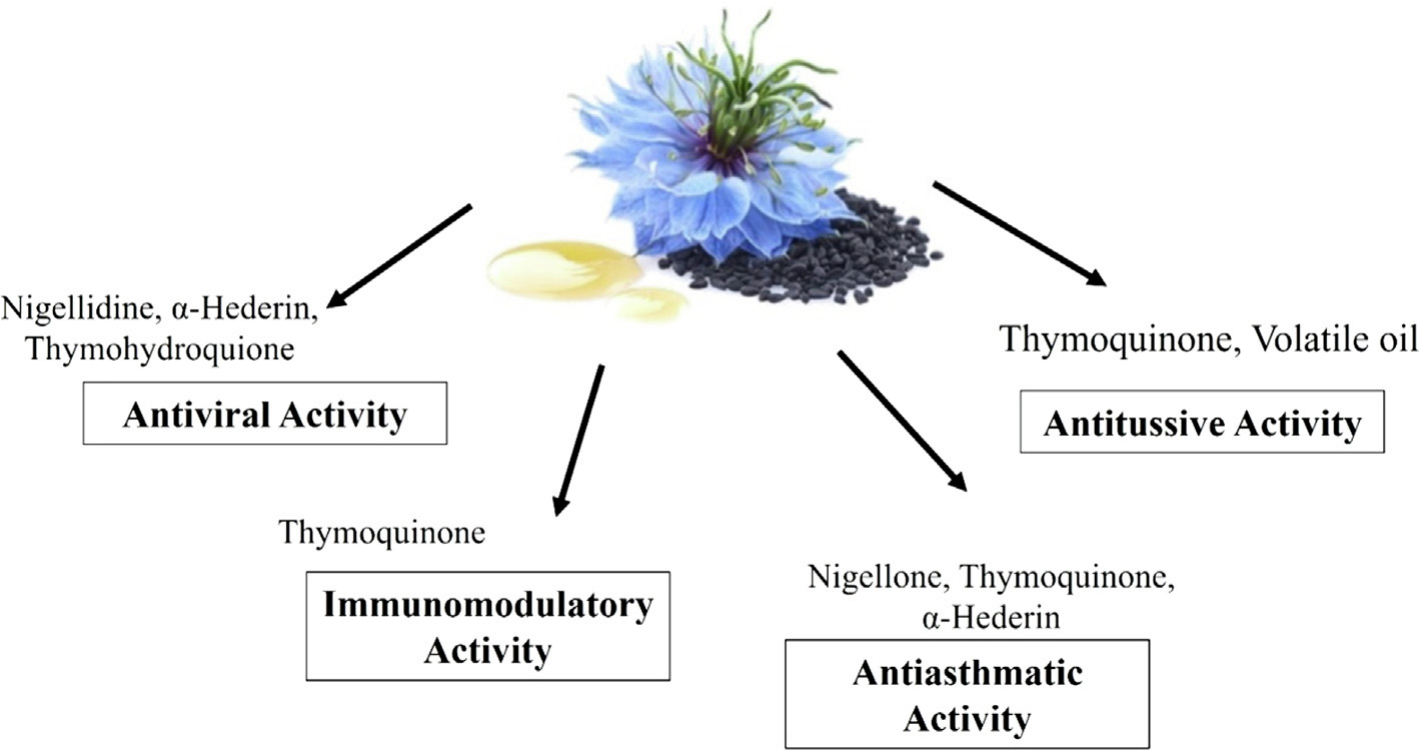
Pictorial representation of the active compounds from *N. sativa.*

Common names of *Nigella sativa* is black cumin, kalonji, Black caraway, Roman coriander, fennel flower, and Habbatus sawda etc*. N. sativa* is the member of Ranunculacea Family (AlAttas et al., 2016). Its plant has a shiny tripartite leaves and slight hairy stem (Srinivasan, 2018). The flower has 5–10 petals with blue, pale and white colours. The fruits are inflated capsules that are further separated into three to seven connected follicles. Every follicle carry several black seeds, each measuring about 1 mm diameter (Gholamnezhad et al., 2016, Mohany et al., 2012) (Fig. 9). It is a dicotyledonous plant and grows in the Western Asia, Eastern Europe, Middle East.

*N. sativa* is the most important plant having religious significance in Muslim culture as well as in christanity (Pendidikan et al., 2020). Furthermore, it is considered as cure of every illness except death in Muslim tradition according to a narration by Prophet Muhammad (PBUH). Traditionally, *N. sativa* has been used in various medicine to treat illnesses such as asthma, colds, headaches and stuffy nose (Mollazadeh et al., 2017). It possess important medicinal properties such as antioxidant, anticancer, immunomodulator, antimicrobial, antiasthmatic as well as bronchodialor (Kooti et al., 2016).

**Fig. 10:**
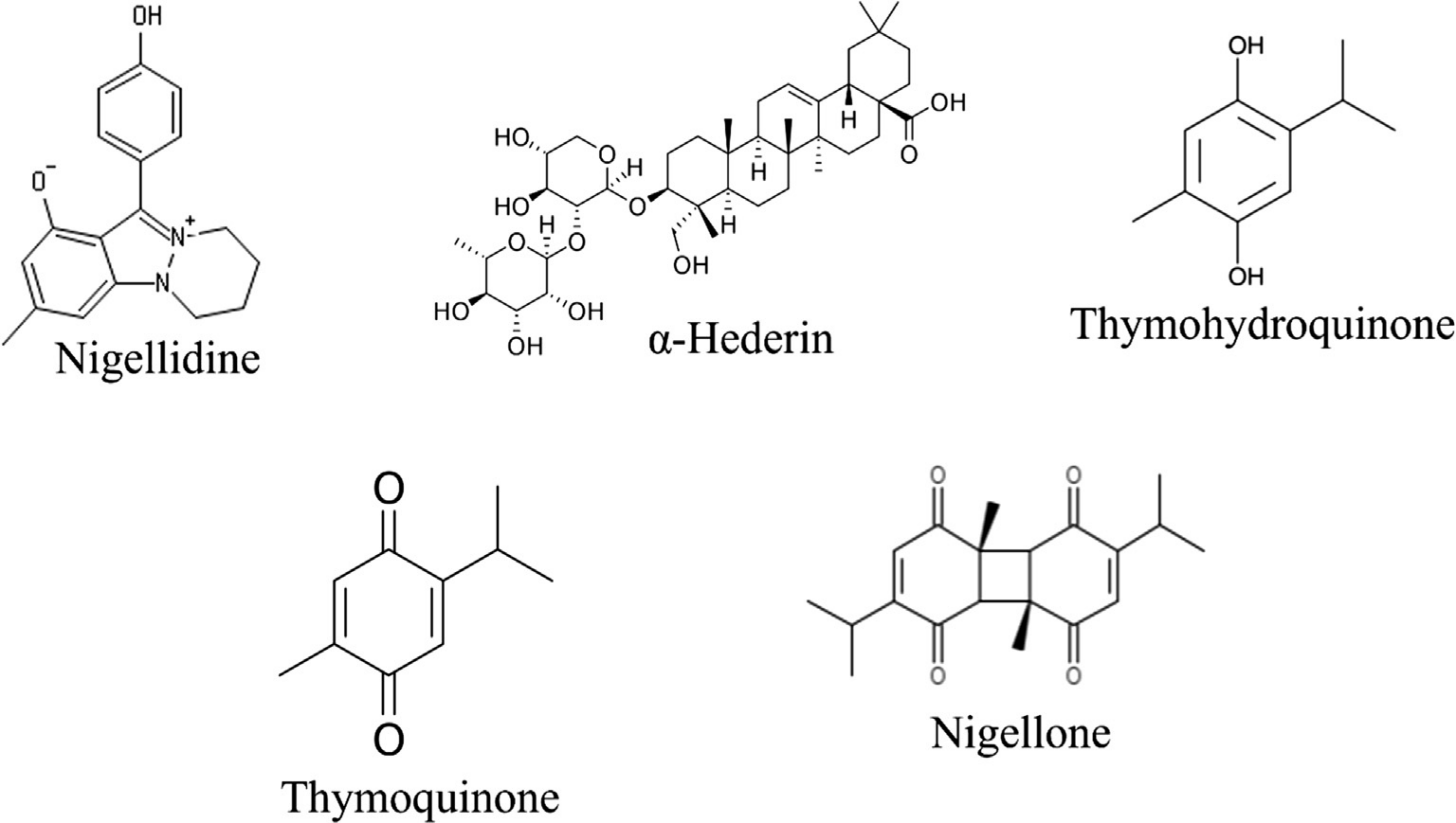
Active flavonoid compounds from *N. sativa.*

The medicinal benefits of *N. sativa* have been documented in Chinese and unani medicine, Ayurveda, and other healing systems (Ahmad et al., 2013).Various bioactive ecompounds including flavonoids, terpenes, tannins, coumarins, phenolic compounds, alkaloids, cardiac glycosides, saponins, fatty acids, volatile oils, terpenes (e.g., TQ), dithymoquinone (DTQ), limonine, p-cymene, indazole alkaloids like nigellidine and nigellicine, and isoquinoline alkaloids including nigellicimine, nigellicimine-*N*-oxide and α-hederin are found in *N. sativa* (Fig. 10) (Akram Khan and Afzal, 2016). Its seeds contain protein, 36–28 % fixed oil, alkaloids, saponins, and 0.4–25 % essential oils. Fatty acids like arachidonic, eicosdienoic, and linoleic acid as well as palmitic, stearic, myristic acid are present in oil (Srinivasan, 2018).

Additionally, carbohydrates, vitamins, lipids, minerals, and proteins containing eight or nine essential amino acids, are among the additional nutritious components present in seeds (Morris and Mohiuddin, 2020). Black cumin oil's main ingredient is p-cymene. Traditionally, it is utilized to treat cough, asthma, bronchitis, pyrexia and flu (Burits and Bucar, 2000). As *N. sativa* possesses antiviral, immonomodulatory, antipyretic, bronchodilatory activities, hence it is speculated that it can be a useful herb for treating people with COVID-19 (Gholamnezhad et al., 2016). Furthermore, bioactive compounds like nigellidine and α-hederin have been considered as powerful SARS-CoV-2 inhibitors (Bibi et al., 2020, Biswas et al., 2021, Salim and Noureddine, 2020).

#### 3.5.1 Antiviral activity

*N. sativa* possess known antiviral activity against some viruses like (MCMV) (Salem and Hossain, 2000), PRSV (Maurya et al., 2005), Avian influenza virus (Salem and Hossain, 2000), Newcastle disease virus (Khan et al., 2018). The antiviral activity is considered due to the increased serum interferon-gamma levels, enhanced suppressor function, increased CD4 count, and increased macrophage numbers. Extract of *N. sativa* contains many important antiviral and antimicrobial active compounds including thymoquinone, p-simen, karvakrol, *trans*-anethole (0.25 % to 2.3 %), 4-terpineol (2.0 % to 6.6 %), and longifolene (Salem and Hossain, 2000). Beta-pinene a monoterpene compound showed antiviral activity against infectious bronchitis virus (Yang et al., 2011). Limonene is also a monoterpenic compound that was analysed against yellow fever virus and tobacco virus (Gómez et al., 2013). Three crucial COVID-19 proteins are papain-like protein, 3C-like protease, and S protein (SP), which are similar to the SARS virus (Bibi et al., 2021).

Molecular docking analysis showed that these proteins acts as potential drug target against *N. sativa* compounds. Nigellidine and α-hederin showed significant binding attraction to above mentioned SARS-CoV-2 proteins (Zhang et al., 2020). With SARS-CoV-2 6LU7, endoribonuclease, RNA-dependent RNA polymerase, SARS-CoV-2 binding domain and human ACE2, thymohydroquinone has moderate docking energies (da Silva et al., 2020). *N. sativa* lowers the coronavirus load in infected Hela cells by upregulating the synthesis of interleukin 8 and downregulating transient receptor potential (TRP) genes such TRPM6, TRPA1, TEPC4, and TRPM7 (Ulasli et al., 2014).

#### 3.5.2 Anti-asthmatic activity

Basophils and mast cells release histamine the nitrogenous compound, producing allergic reactions associated with brochial asthma (Saadat et al., 2015). Nigellone from *N. sativa* showed antiasthmatic activity by inhibiting the histamine release from the mast cells (Mathur, 2011). In an animal model of allergic asthma, alpha-hederin of *N.sativa* improved tracheal responsiveness and had considerable anti-inflammatory effect by lowering histamine and leukotriene production while boosting PGE2 from mast cells and perfused lungs (Akram Khan and Afzal, 2016, AlAttas et al., 2016, Ikhsan et al., 2018). In asthma, leukotrienes may act as inflammatory mediators. Thymoquinone inhibit the formation of leukotrienes and improve choking, pulmonary function testing, and many asthma symptoms (Srinivasan, 2018).

#### 3.5.3 Immunomodulatory activity

*N. sativa* seeds boost immunity by rising up T4:T8 ratio and activating the natural killer cell (Ahmad et al., 2021). The oil, as well as the active ingredient in Thymoquinone, has immunomodulatory properties, boosting immunological responses in T lymphocytes and natural killer cells (Salem, 2005). Thymoquinone's immunomodulatory effects on pesticide-induced immunotoxicity in male albino rats were examined by Mohany and colleagues (Mohany et al., 2012). Asthma inflammation is well immunomodulated by thymoquinone extract, which inhibits IL-2, IL-6, and PGE2 in T cells and IL-6 AND PGE2 in monocytes (Koshak et al., 2018).

#### 3.5.4 Antitussive activity

Experimental study on guinea pigs proved that *N. sativa* showed antitussive activity. According to Mahfouz and Dakhakhnsy (1960), the essential oil of *N. sativa* protects guinea pigs from histamine-induced bronchospasm. Thus, volatile oil increases intratracheal pressure and respiratory rate of pigs. In another study, plant extract aerosols were found to be as effective as codeine at reducing the number of coughs brought on by citric acid aerosol (Pattanayak and Sunita, 2009). Thymoquinone present in *N. sativa* showed antitussive properties due to its brochodilatory, anti-inflammatory actions which are mediated by opioid receptors (Dey et al., 2022, Hosseinzadeh et al., 2008). The pharmacologiacally important compunds of *N. sativa* has been presented in Table 5.

### 3.6 Cassia angustifolia

**Fig. 11:**
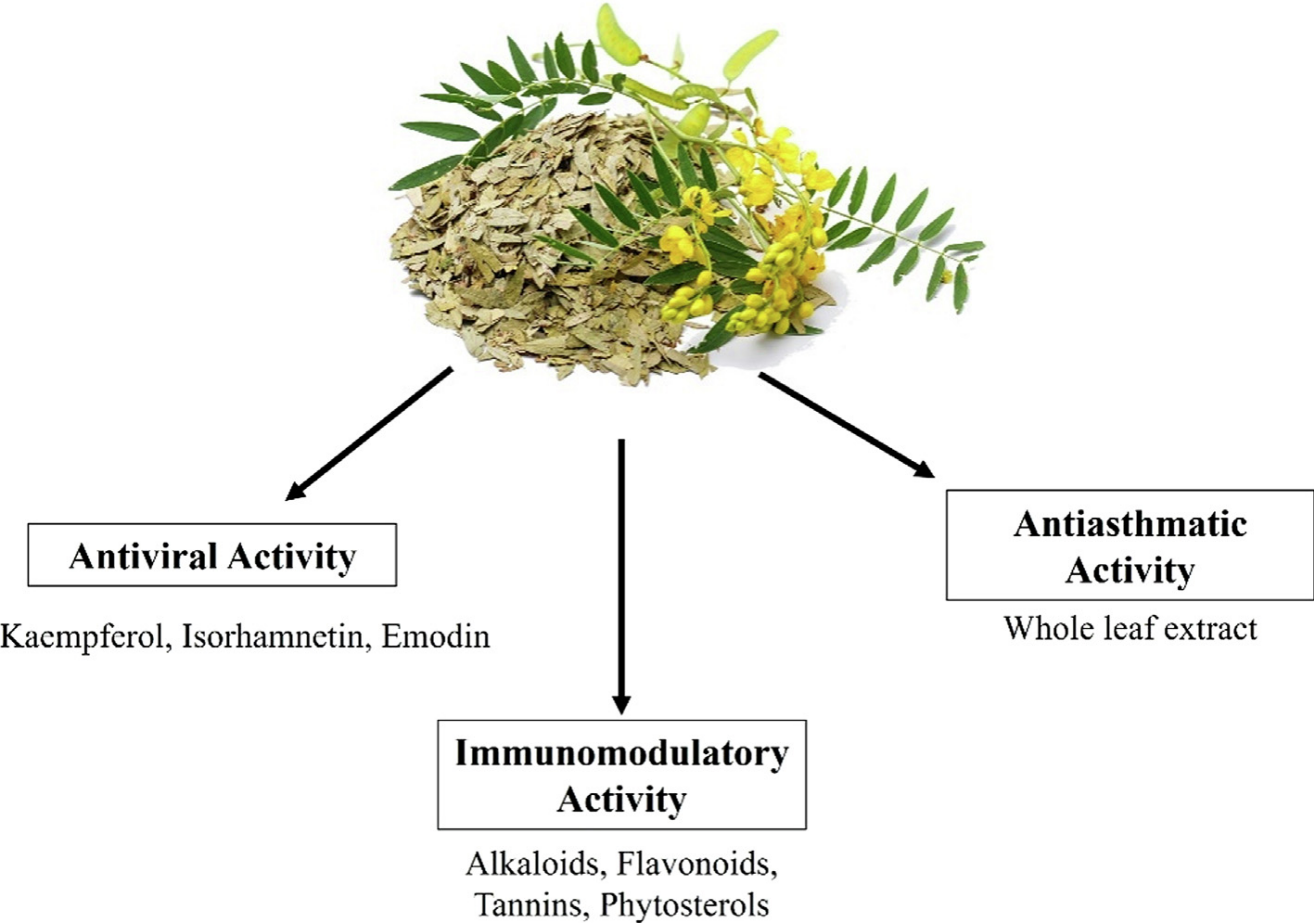
*C. angustifolia* and its active compounds.

The common name of *Cassia angustifolia* is senna drought resistant herb. The plant has 1 m in height with ascending branches. The flowers are arranged in auxiliary erect, racemes and bright in colour. Greenish brown pods with 5–7 smooth, obovate dark brownseeds are 1.4 to 0.8 in. broad. Leaves have usually 5–8 leaflets. Overall, glabrous and lanceolate,the fully matured leaflets ranges from blush green to pale yellow colour (Shazia Sultana, 2012) (Fig. 11). Cassia is a largest genus in the family Leguminosae, with over 500 flowering plants species (Lodha et al., 2010). Caesalpiniaceae is Cassia family species. Caesalpiniaceae is subfamily of Caesalpinioidae, the large family of Leguminosae (Gagnon et al., 2016).

*C. angustifolia* has a significant medicinal importance in Muslims ethnobotanical culture when identified in and around blessed Makkah city, the capital of the Hijaz province. The Holy Prophet Muhammad (PBUH) was the first to use the plant as a herbal medicine (Ahmad et al., 2010, Bibi et al., 2022) and narrated “*If any treatment against death, it would be Senna, the blissful, the graceful one*“ (Maciej Serda, 2013). Nowadays, senna is grown globally in India, Pakistan, Sudan, China, Europe etc. It is sold in herbal shops in Pakistan, India, and Arabia under the names senna or sana makki. It is broadly used in traditional Chinese, Indo-Pakistani, and African medicine, and it's also used in the allopathic medical system in Western countries (Kumar et al., 2020).

**Fig. 12:**
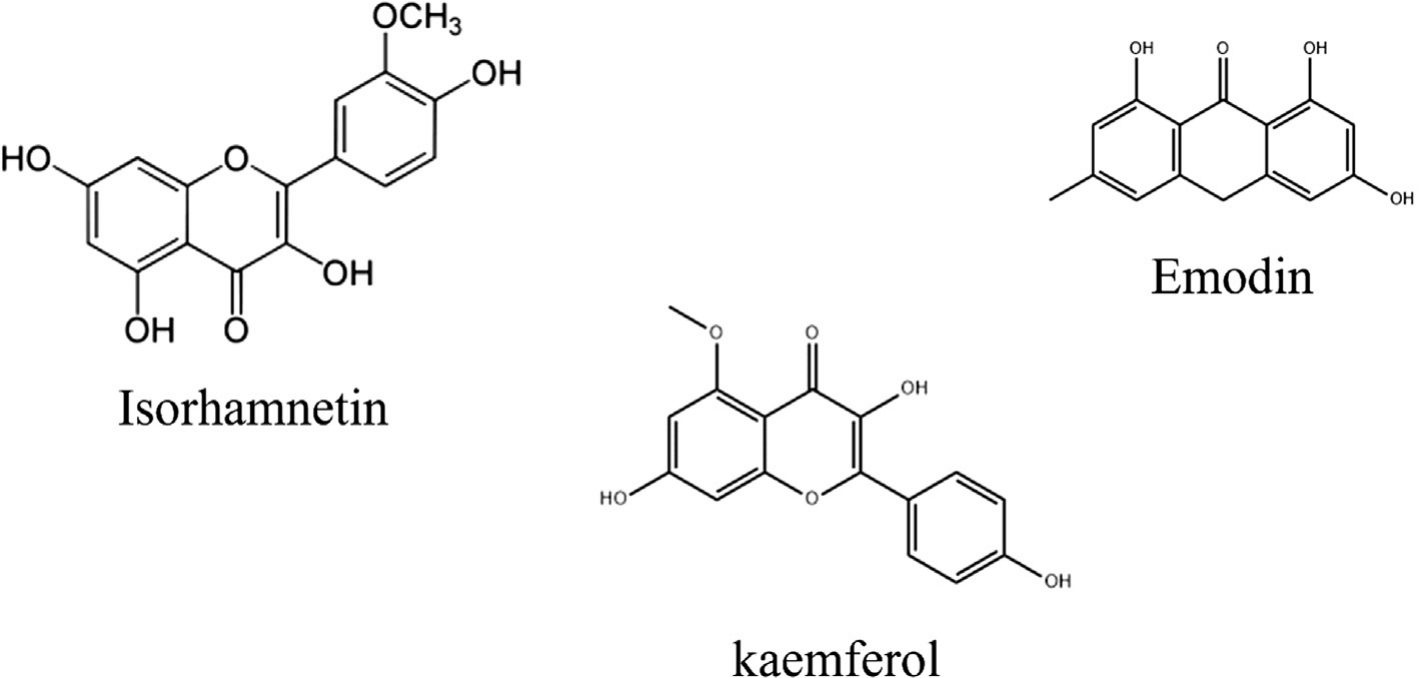
Chemical compounds from *C. angustifolia.*

Senna is commonly used to cure constipation, asthma, digestive disorders, fever, bronchitis, ameboid dysentery and hemorrhoids (“Recent Advances in the Phytochemistry of Some Medicinally Important Cassia Species: A Review - Volume 2, No. 3, July 2013 - IJPMBS,” n.d.). The laxative quality of senna is due to two glycosides such as sennoside A and sennoside B, where as sennoside C and D also present in plant. Apart from sennoside, glycosides of anthraquinones, chrysophenic acid, rhein are also present in cassia pods and leaves (Fig. 12) (Le et al., 2021). The flavonoids kaemferol, kaemperin, rhein and isorhamnetin are also present in cassia (Nair et al., 2002). Additionally, butanolic triterpenoid glycoside has been isolated from seeds extracts (Khan and Srivastava, 2009). Various compounds such as nigrin, cathartic acid, Rhamnetin, *gluco*-sennin, emodin and salicyclic acid are also extracted from cassia (Sorina et al., 2017) and could be used as an alternative medicine to treat the symptoms of COVID-19 (Biswas et al., 2021, Khan et al., 2022).

#### 3.6.1 Antiviral activity

Previously, kaempferol showed antiviral activity angainst PRV (Pseudorabies virus). Kaempferol inhibits virus replication in brain of PRV-infected mice and shows effectiveness in delayed or inhibition of the clinical symptoms (Zhao et al., 2018). Similarly, Isorhamnetin may interact with the SARS-CoV-2 functional ACE2 receptor, preventing viral entrance and infection in human cells that express ACE2. Isorhamnetin is considered as a novel potential medication for COVID-19 management as an ACE2-protein interaction inhibitor (Yang et al., 2020). Moreover, emodin showed antiviral activity against coronavirus such as SARS-CoV-2 recently (Bibi et al., 2022, Zhou et al., 2020b). Emodin inhibits casein kinase-2, that is used by several viruses to phosphoryate proteins necessary for their life cycle (Battistutta et al., 2000).

#### 3.6.2 Immunomodulatory activity

Methanol has been found to be the most effective solvent for extracting several secondary metabolites from *C. angustifolia*. Alkaloids, flavonoids, tannins, phytosterols, and other metabolites are powerful antioxidants and are extracted in methanol. They reduce immune cell damage by inhibiting the excessive production of reactive oxygen species. Ag, Mn, Ca, Mg, Zn, Cu, Na, k, vit E, and other minerals that are also present in the plant (Basak and Gajbhiye, 2018, Parveen et al., 2016). Almost all chemicals that potentially contribute to the reported effects on haematological parameters were extracted using methanol. Bioactive substances, particularly Ag and vit E, were found to becrucial for the formation and maturity of the immune system by promoting hemopoiesis cellular components.

*C. angustifolia* contains polyunsaturated fatty acids and trace elements that are essential for neutrophil function in cell-mediated immune response. Zinc plays important role incytosolic superoxide dismutase activity, that aid neutrophils to live longer by inhibiting oxidation processes (Maggini et al., 2007). Alcoholic extract of *C. angustifolia* stimulates serum protein production, which is vital in the body's defence mechanism (Ilavarasan, 2001). Copper is essential for enzyme cerruloplasmin which is involved in humoral immunity (Moyo et al., 2011).

#### 3.6.3 Antiasthamatic activity

*C. angustifolia* enhances oxygen absorption for the respiratory system by improving mucous outflow of lungs and lessen mucus in the airway. Cough medicine and bronchitis treatment are both made with senna leaf extract^.^ (Kim and Criner, 2013). The important compounds found in *C. angustifolia* are summarized in Table 6.

## 4 Discussion

COVID-19 is amongst one of the most emerging health anxieties having a drastic influence on human life. Despite the hazardous caused by COVID-19, there is no specific drug for treatment and long lasting cure except for the few vaccines. In past, the tremendous role of herbal preparations addressed many serious ailments and infectious diseases, so the case of COVID-19 management in present era.

To our best knowledge, this is first study summerizing the medicinal benefits of Traditional Arabic or Islamic medicinal (TAIM) plants used in ancient Muslim ethnobotanical culture. Based upon present data, it is tempting to conclude that TAIM plants could be an alternative and potential herbal medicine to answer the various questions in COVID-19 management. The antiviral role of these plants is evident through various scientific studies. The ability of these bioactive compunds (present in TAIM plants) to interfere with the fusion mechanism by binding with the spike protein of virus and inhibiting the SARS-CoV-2 replication. The inhibition of reproduction of virus ultimately leads toward the modified molecular pathways to effectively manage the COVID-19.

Additionally, various parts of these plants could be used as an introductory adjuvant components for COVID-19 treatment to relief the most common symptoms like fever and cough produced by their anti-inflammatory effect. Moreover, many herbal drugs used to boost immune system including *Allium sativum, Althea officinalis, G. glabra*, *ginseng* and *Thymus vulgaris* may be used as potential preventive measures against COVID-19. These herbs may be independently used as an alternative therapy, or as complimentary medicine to treat COVID-19. Thus, as an alternative measures TAIM plants and their bioactive fractions could provide a synthetic route in prevention of COVID-19.

## 5 Conclusion

In this study, we have reported the TAIM plants having evident antiviral activity and can be used as therapeutics against COVID-19. The study provides refrences on the traditional usage of six important medicinal plants including *A. vera*, *O. europeae, T. foenum-graecum, F. carica, N. sativa,* and *C. angustifolia* as antiviral therapy. A considerable proportion of the world still use these plants as a part of their tradition, culture or due to religious significance. Although the medicinal plants could provide promising synthetic route for pre-clinical trials but no significant evalution of these bioactive compounds against COVID-19 has been conducted. Therefore, further studies are to be conducted to assess the safety profiles and effectiveness of these plants. Moreover, evident based, more rigorous high quality human trials are suggested to evaluate the therapeutic potential of TAIM plants for the clinical management of COVID-19.

## 6 Institutional Review Board Statement

Not applicable.

## 7 Informed Consent Statement

Informed consent was obtained from all subjects involved in the study.

## Declaration of Competing Interest

The authors declare that they have no known competing financial interests or personal relationships that could have appeared to influence the work reported in this paper.
